# Visual Perception and Autonomous Navigation Control for Orchard Robots: From Scene Understanding to Closed-Loop Motion Execution

**DOI:** 10.3390/s26206402

**Published:** 2026-10-10

**Authors:** Yunfei Wang, Xiang Dong, Zhenlei Zhang, Zhihao Zhang, Weidong Jia

**Affiliations:** 1 School of Agricultural Engineering, Jiangsu University, Zhenjiang 212013, China; 2112416035@stmail.ujs.edu.cn (Y.W.); dongxiang@ujs.edu.cn (X.D.); 2222416001@stmail.ujs.edu.cn (Z.Z.); 2222616088@stmail.ujs.edu.cn (Z.Z.); 2 Key Laboratory of Plant Protection Engineering, Ministry of Agriculture and Rural Affairs, Jiangsu University, Zhenjiang 212013, China

**Keywords:** orchard robot, visual navigation, scene understanding, navigation-line extraction, visual SLAM, sensor fusion, path tracking, closed-loop control

## Abstract

Autonomous navigation is essential for orchard robots performing spraying, weeding, inspection, harvesting, and transport, yet semi-structured orchards combine repeatable tree-row geometry with canopy occlusion, uneven illumination, weak road boundaries, terrain variation, and intermittent global positioning. This review examines orchard visual navigation through a control-oriented chain of scene information, perception and localization, navigation-reference construction, motion execution, and closed-loop evaluation. Particular emphasis is placed on orchard-specific interfaces: repeated row geometry can stabilize reference extraction but can also create perceptual ambiguity; canopy occlusion can simultaneously degrade visual features and GNSS availability; and headland turning requires explicit switching among row following, turning, relocalization, and task resumption. Passable areas, tree-row/canopy structure, and obstacle/end-of-row semantics are compared according to the control variables they generate, including drivable corridors, lateral and heading errors, curvature, safety clearance, event states, and tracking targets. Traditional vision, deep-learning detection and segmentation, depth vision, visual SLAM, multi-sensor fusion, and path-tracking control are synthesized in terms of operating conditions, failure modes, and downstream control effects. The review further analyzes perception-to-control error propagation, end-to-end latency, cross-season generalization, and the mismatch between image metrics and field navigation performance. A unified perception–representation–localization–planning–control–evaluation framework and full-chain benchmark protocol are presented.

## 1. Introduction

Recent reviews of agricultural deep learning, visual navigation, and motion planning show that autonomous mobility is becoming an enabling layer for plant protection, mowing, scouting, harvesting support, and material transport rather than an isolated steering function [1,2,3]. In orchards, however, this mobility must operate in a semi-structured environment: repeated trunks and tree rows provide useful geometric regularity, whereas canopy growth, pruning, ground cover, illumination, and terrain continually alter the appearance and physical clearance of the inter-row corridor. Consequently, orchard navigation cannot be reduced to either structured-road lane keeping or open-field GNSS guidance.

Reviews of facility robots, transfer learning in agriculture, and automated agricultural machinery consistently identify localization reliability, domain shift, and heterogeneous sensing as major barriers to field deployment [4,5,6]. In orchards, GNSS/RTK can provide accurate global position in open areas but is vulnerable to attenuation, multipath, and intermittent loss beneath canopies and trellises. Vision complements global positioning by directly observing local motion constraints—drivable ground, trunks, row structure, obstacles, and task targets—but its performance is affected by illumination, occlusion, vibration, and seasonal appearance changes. The practical problem is therefore not to identify a universally superior sensor, but to maintain a control-relevant local representation as sensing conditions change.

Against this background, orchard navigation has expanded from color- and edge-based image processing to deep detection and segmentation, depth vision, visual odometry, simultaneous localization and mapping (SLAM), and multi-sensor fusion; path planning and tracking have likewise progressed from geometric rules to optimization-based, robust, and learning-assisted control [7,8,9,10]. Planning research in semi-structured environments further provides transferable principles for handling incomplete boundaries and locally constrained motion [11]. Existing reviews have established useful taxonomies of agricultural robots, orchard machinery, motion planning, and path-tracking control, but perception outputs, navigation-reference construction, and vehicle execution are still frequently treated as separate technical layers.

For orchard robots, this separation obscures the dominant failure mechanisms. High mean average precision (mAP) does not guarantee a stable row centerline; a smooth planned path may be referenced to a biased pose; and a high-performance controller can accurately follow an incorrect reference. Recent agricultural-navigation and mobile-robot reviews therefore increasingly emphasize coupling among sensing, planning, and tracking, together with sensor reliability, uncertainty, and closed-loop validation [12,13,14,15].

Evaluation should therefore extend beyond isolated recognition metrics to the complete information-to-control chain: which environmental variables are observed, how they are converted to metric geometry, how references and constraints are generated, and how uncertainty and latency affect vehicle response. Recent crop-row, autonomous-tractor, sensor-fusion, and orchard-navigation reviews support this system-level perspective [16,17,18,19,20], but an explicit control-oriented synthesis of visual perception remains limited.

The coupling among these modules is specifically shaped by orchard structure. Repetitive tree rows provide a strong prior for centerline and heading estimation, but the same repetition can reduce place distinctiveness and aggravate data association or SLAM ambiguity. Dense canopies can occlude trunks and reduce image contrast while simultaneously attenuating or multipathing GNSS signals, so perception and localization may degrade together rather than independently. At row ends, the controller must leave a locally constrained corridor, execute a headland maneuver, reacquire the next row, and restore the task state without a discontinuous reference jump. Intermittent GNSS availability therefore changes not only the localization source but also the confidence, reference frame, and mode-transition logic passed to planning and control. These coupled effects distinguish orchard navigation from a generic mobile-robot pipeline and motivate uncertainty-aware interfaces between modules.

To improve the transparency of literature selection, this review adopted a structured search strategy focusing primarily on studies published from January 2018 to September 2026, while retaining selected earlier studies when necessary to trace the origins of key navigation, planning, or control methods. The main sources were Web of Science Core Collection, Scopus, IEEE Xplore, ScienceDirect, and Google Scholar. Search terms covered orchard/agricultural robots, visual navigation, row following, drivable areas, tree-trunk detection, semantic segmentation, RGB-D/stereo vision, LiDAR, visual SLAM, GNSS/IMU fusion, path planning, path tracking, and autonomous control. Studies were considered when they were directly relevant to orchard autonomous navigation and provided sufficient methodological or evaluation information; studies lacking a navigation context or adequate methodological detail were excluded. Candidate studies were first screened by title and abstract and subsequently assessed through full-text review for relevance to orchard navigation.

Accordingly, this review uses a control-oriented lens to connect visual scene understanding with executable orchard navigation. The principal contributions are:

- Orchard observations are organized by navigation function—drivable-space constraints, structural landmarks, and action-relevant semantics—and each category is linked explicitly to the control variables it generates.
- Perception and localization methods are compared not only by recognition accuracy but also by operating conditions, dominant failure modes, temporal stability, metric output, and suitability for downstream planning and control.
- The transformation from perception to reference construction, path tracking, headland transition, and task execution is treated as a continuous information-to-action chain, with specific attention to error propagation and latency.
- A full-chain evaluation protocol is formulated around reference bias and jitter, localization drift, tracking error, recovery time, human intervention, row-transition success, and task-completion rate.

## 2. Orchard Scene Information Required for Visual Navigation

### 2.1. Passable Areas and Road Boundaries: The Primary Spatial Basis for Feasible-Corridor Construction

Passable-area and road-boundary perception provides the primary local motion constraints for orchard robots. Unlike structured roads, orchard alleys may consist of bare soil, grass-covered surfaces, wheel tracks, dappled shadows, or weak-texture corridors defined only indirectly by the two tree rows. Consequently, a binary road/background mask is insufficient for closed-loop navigation. More useful outputs include the drivable region, left and right safety boundaries, corridor width, local heading, and associated confidence. Once transformed into the robot frame, these quantities generate signed lateral error, heading error, left/right clearance, and an uncertainty-qualified corridor that can be consumed by path planning, speed scheduling, and steering control [21,22,23].

**Figure 1:**
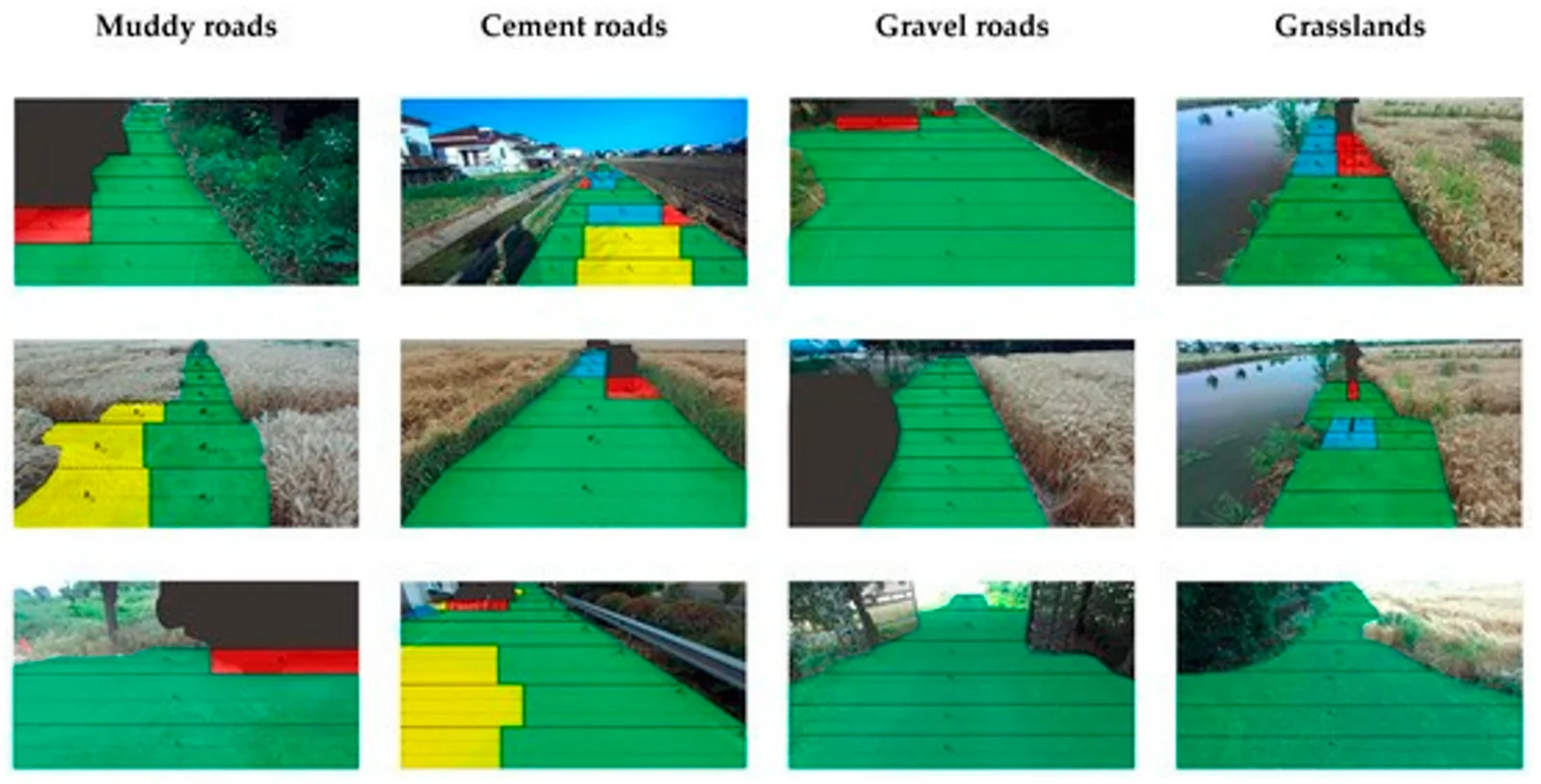
Passable-area evaluation results for agricultural roads, illustrating the conversion from visual road understanding to a navigation-safe driving region (reprinted from Ref. [30] under the Creative Commons Attribution 4.0 International license (CC BY 4.0)).

Representative passable-area studies spanning semantic boundaries, lateral-distance measurement, passability assessment, disturbance-robust guidance lines, clustering, low-light segmentation, and route extraction are represented in Refs. [24,25,26,27,28,29,30]. Chronologically, Chen et al. (2021) converted a visually detected harvesting boundary into a metric lateral distance using a dynamic region of interest [24]. In 2024, Wang et al. used semantic segmentation for harvesting-boundary recognition [25], Liu et al. extracted crop-free ridge guidance lines from stable machine-vision geometry [23], and Zhong et al. improved U-Net-based path recognition for low-light environments [26]. In 2025, FSA-YOLOP explicitly evaluated guidance-line robustness under dim illumination, reflection, missing lines, and occlusion [27], while Fang et al. addressed weak road boundaries through DBSCAN-MFI clustering [28] and Xu et al. converted U-Net segmentation into a continuous orchard navigation route [29]. Zhang et al. (2026) further extended the output from boundary geometry to road passability through improved YOLOv5s and multi-factor fusion [30]. The sequence reflects a transition from visible-feature extraction to control-oriented traversability representation. Representative passability results are shown in Figure 1.

Table 1 compares this development from direct geometric or segmentation outputs toward control-ready passability, with emphasis on operating conditions, suitable use, engineering strengths, and dominant failure modes.

Complementary orchard-road segmentation, ridge-tracking, and dynamic replanning studies are summarized in Refs. [31,32,33], providing additional evidence on scene-specific road extraction, filtered relative tracking, and replanning under changing obstacles.

Overall, passable-area perception has shifted from detecting a visible boundary to constructing a locally drivable corridor with explicit safety meaning. Recent crop-row reviews and orchard navigation-line studies further emphasize that segmentation accuracy alone does not determine navigation quality; reference continuity, actual vehicle width, and uncertainty propagation are equally important for closed-loop operation [34,35].

The principal limitation is that visual appearance is not in one-to-one correspondence with physical traversability. Grass cover, wheel tracks, water reflections, canopy shadows, and seasonal ground changes may substantially alter image statistics without changing whether the robot can pass safely. A more robust corridor representation may therefore retain boundary confidence, temporal persistence, and a geometric safety margin so that the controller can reduce speed, enlarge clearance, or switch to structural landmarks when visual evidence degrades. Evaluation can correspondingly extend from frame-wise mIoU or detection accuracy to reference bias, jitter, corridor continuity, and closed-loop traversal success.

### 2.2. Trunks, Tree Rows and Canopy Structure: Natural Landmarks for Relative Positioning and Row Following

Trunks, tree rows, and canopy boundaries provide relatively persistent structural priors in orchard environments. When road texture is ambiguous or GNSS quality deteriorates beneath the canopy, these features can generate row direction, relative lateral position, heading error, local corridor geometry, and landmark confidence. Navigation-oriented structure perception therefore requires not only semantic detection but also metric landmark coordinates, left/right association, robust row fitting, and temporal consistency [36,37,38]. Representative orchard studies covering trunk detection, metric localization, and field navigation are summarized in Refs. [39,40,41,42,43,44,45,46].

**Figure 2:**
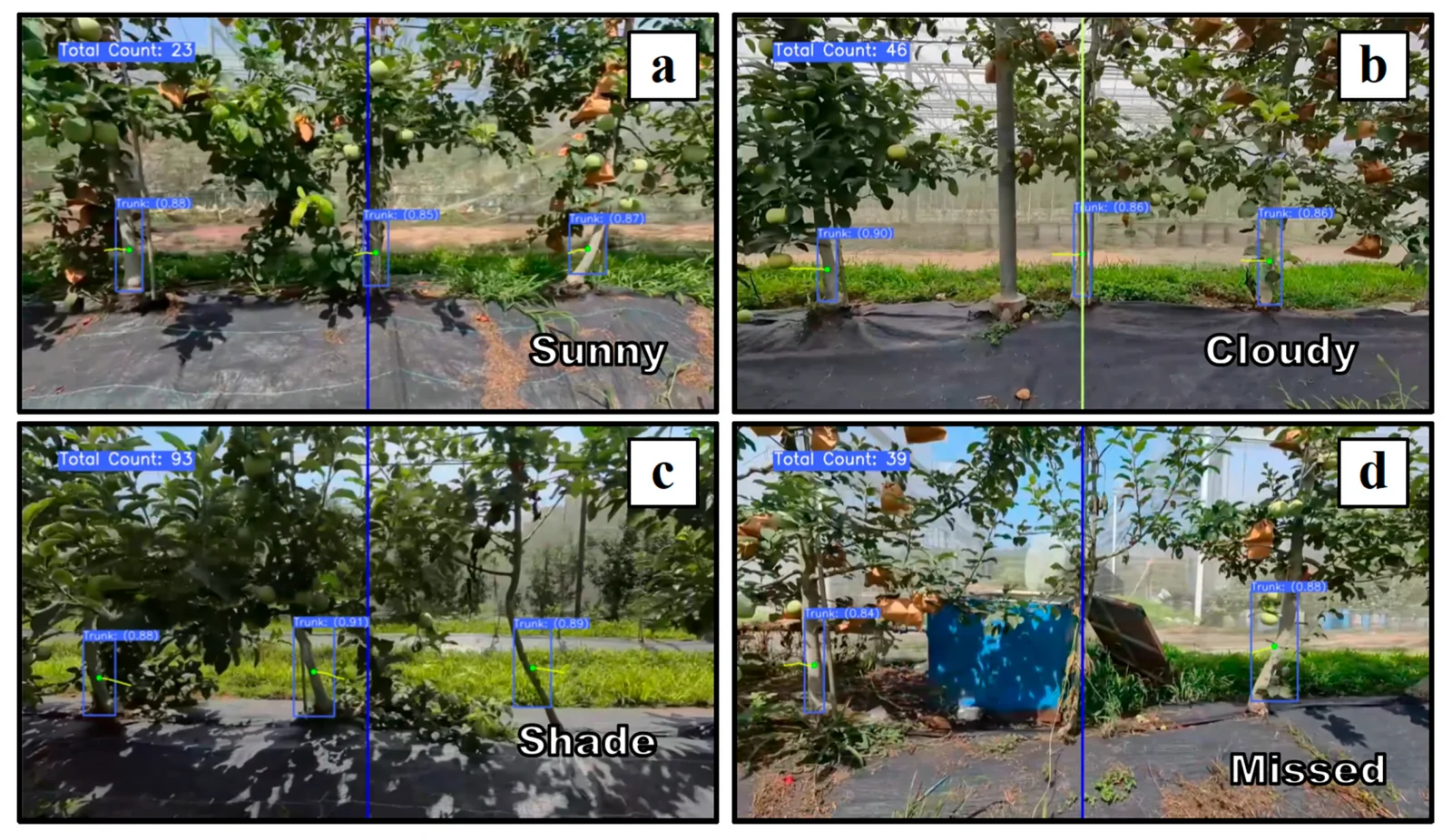
Dynamic spindle-shaped trunk detection under representative orchard illumination conditions: (**a**) sunny; (**b**) cloudy; (**c**) shaded; (**d**) missed detection (reprinted from Ref. [45] under the Creative Commons Attribution 4.0 International license (CC BY 4.0)).

The development can be understood chronologically. Radcliffe et al. (2018) demonstrated direct orchard guidance from canopy–sky geometry [39], followed by Blok et al. (2019), who validated 2D-LiDAR probabilistic localization in a commercial apple orchard [40]. Opiyo et al. (2021) then coupled machine-vision medial-axis extraction with fuzzy steering control, directly linking visual path geometry to vehicle motion [41]. In 2023, Huang et al. integrated trunk perception and depth estimation with DWA-based local navigation [42], whereas Gu et al. converted binocular trunk observations into lateral and heading states relative to fruit-tree rows [43]. More recent RGB-D and instance-segmentation studies have strengthened metric landmark localization [37,44], and Zheng et al. (2026) evaluated spindle-shaped trunk detection under sunny, cloudy, shaded, and missed-detection conditions [45]. Collectively, the progression is from appearance cues to metric structural states that can be consumed directly by planning and control. Representative dynamic trunk-detection results are shown in Figure 2.

Table 2 compares representative landmark-based methods by operating condition, control-relevant output, engineering strength, and dominant limitation. Across the studies, a consistent pattern emerges: canopy or trunk semantics become substantially more useful for navigation once metric scale, row association, and temporal persistence are recovered.

From the control-chain perspective, tree landmarks pass through semantic detection, metric localization, left/right association, robust fitting, and lateral/heading-state estimation before they become an executable navigation reference. Systematic bias at any stage can shift the reconstructed row axis; evaluation therefore benefits from landmark-coordinate stability, the number of valid landmarks, fit residuals, and continuity of the final row reference.

The main limitations arise from incomplete visibility and the non-rigid nature of orchard landmarks. Foliage can intermittently occlude trunks, trellis posts may be confused with stems, and canopy boundaries vary with wind, pruning, and season. A degradation-aware row model may retain observation confidence, left/right association status, and the number of supporting landmarks, allowing fusion and control weights to be adjusted before a single-frame error produces an abrupt steering command.

### 2.3. Obstacles, Row Ends and Task Semantics: Action-Relevant Scene Understanding for Safe Navigation

Safe orchard navigation requires more than stable row following; the robot must determine which scene elements should modify motion. Flexible vegetation, rigid obstacles, people, irrigation infrastructure, and row-end regions may occupy similar image space but require different responses. The control-relevant outputs are obstacle class, metric position, occupied extent, relative motion, confidence, free-space clearance, and discrete events such as “row end detected.” These variables feed local collision constraints, speed commands, avoidance priorities, and the supervisory state machine that switches among row following, stopping, headland turning, and re-entry [47,48,49].

**Figure 3:**
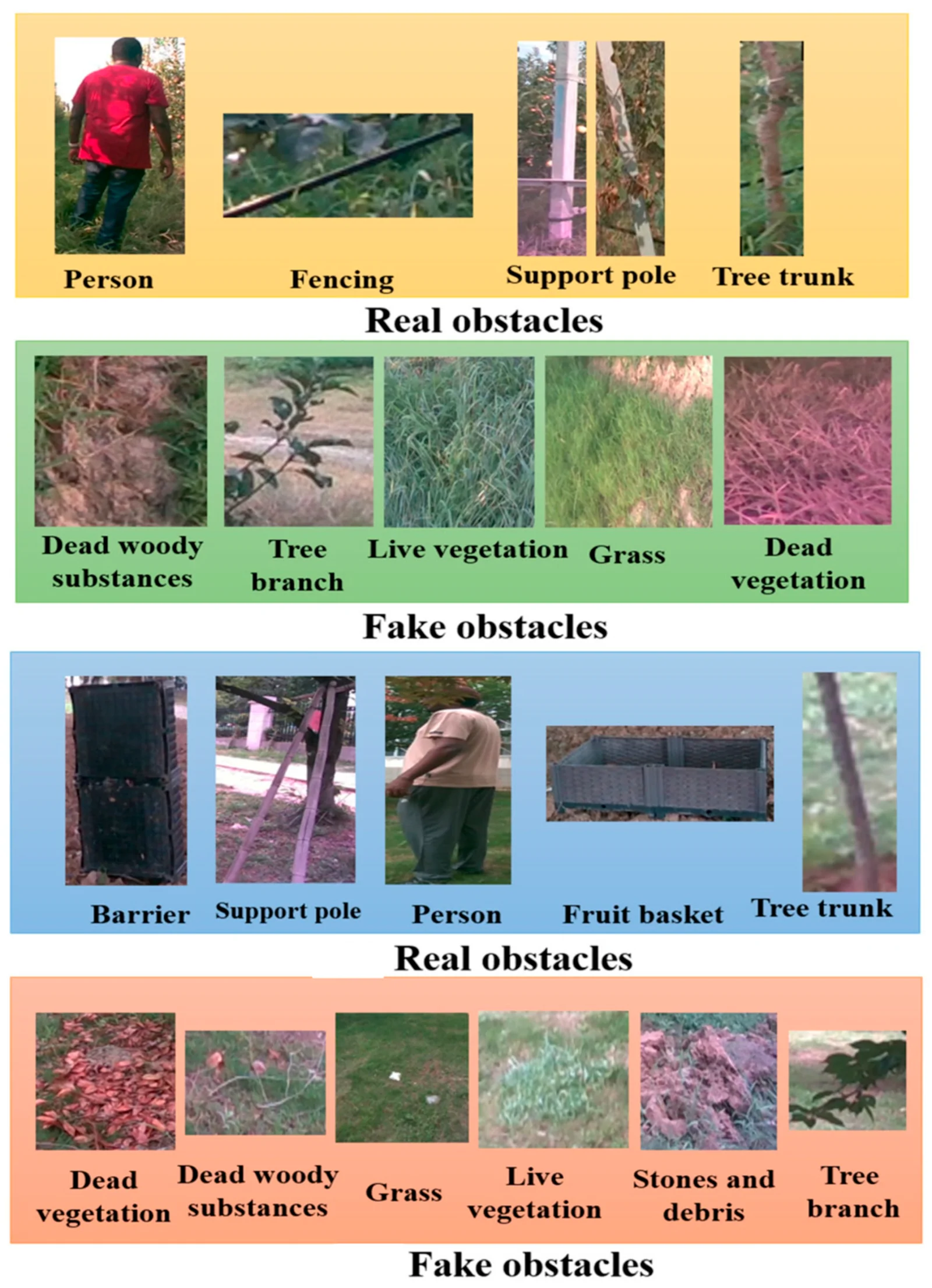
Real/fake obstacle classification for autonomous orchard navigation (reprinted from Ref. [48] under the Creative Commons Attribution 4.0 International license (CC BY 4.0)).

Representative studies show the transition from detecting objects to estimating how they alter feasible motion. Inoue et al. (2022) combined machine vision and obstacle avoidance for an orchard mowing robot, demonstrating that narrow tree clearance must be coordinated with local avoidance [50]. Later work expanded scene interpretation toward action-relevant semantics: Syed et al. separated “real” obstacles from vegetation or structures that should not trigger the same response [48], Navone et al. coupled depth semantic segmentation with GPS-free row following [51], and Wang et al. mapped obstacle observations directly into velocity and trajectory choices through an improved dynamic-window approach [52]. Representative real/fake obstacle classification is shown in Figure 3.

Beyond the core orchard studies, A*, DWA, artificial-potential-field, ant-colony, and hybrid planners convert occupancy evidence into inflated obstacle boundaries, collision risk, and feasible velocity sets [53,54,55,56,57,58]. Additional semantic and planning examples are summarized in Refs. [59,60,61,62]. Table 3 compares these approaches by the decision variable they generate, the conditions in which they are useful, and the failure mode most likely to affect downstream motion.

Overall, a control-oriented obstacle representation should retain at least class, spatial position, occupied extent, confidence, and motion state, whereas row ends are more appropriately represented as events that trigger the transition from row following to headland turning and re-entry into the next row. Representative studies on obstacle perception, free-space understanding, and local avoidance are summarized in Table 3.

The major unresolved problem is semantic ambiguity and inter-module coupling. Flexible branches, shadows, trellis posts, suspended irrigation lines, and dense weeds may look similar but demand different control actions. Moreover, an avoidance module designed independently from row following can destabilize the primary row reference. A compact semantic set with explicit control meaning, shared drivable-corridor representation, and common risk/task states may reduce conflicts between row-following and local-avoidance modules.

## 3. Key Perception and Localization Technologies

### 3.1. Traditional Machine Vision and Geometric Processing: Interpretable Extraction of Navigation References

Traditional machine vision and geometric processing remain essential because the variables consumed by a controller are geometric even when a neural network is used at the front end. Color-space transforms, vegetation indices, thresholding, morphology, Hough transforms, vanishing-point analysis, least-squares fitting, and RANSAC provide low computational cost, clear physical interpretation, and convenient integration with vehicle kinematics. Their weakness is sensitivity of handcrafted appearance features to illumination, shadows, weeds, and seasonal change. A productive role for geometry is therefore as structured post-processing and as the control interface of modern perception systems [63,64,65].

**Figure 4:**
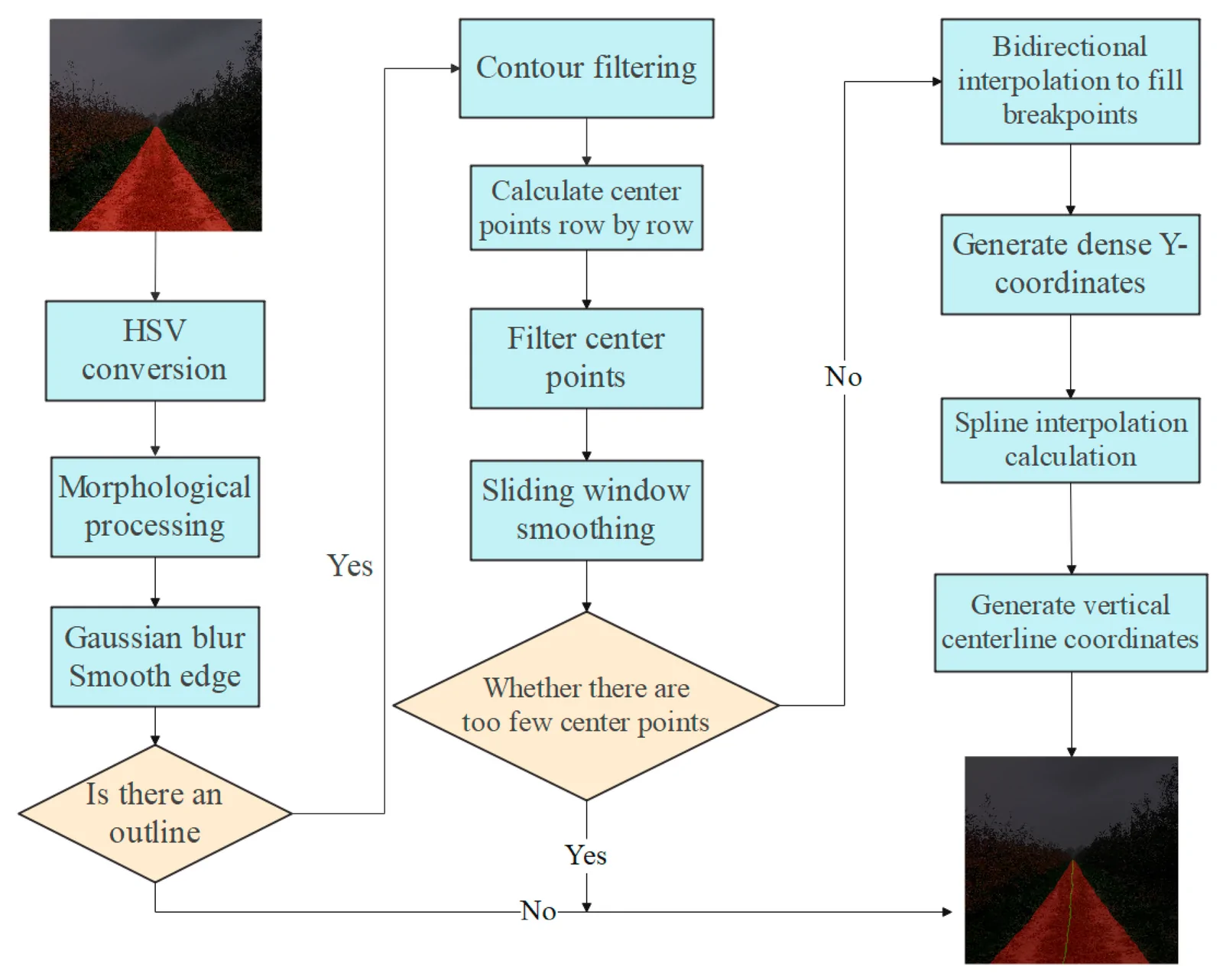
Geometric route-calculation process following drivable-area perception in an orchard (Reprinted from Ref. [29] under the Creative Commons Attribution 4.0 International license (CC BY 4.0)).

Chronologically, the field has moved from direct geometric guidance toward learned perception constrained by explicit geometry. Chen et al. (2021) converted an image boundary into physical lateral distance [24]; Zhou et al. (2022) detected orchard trees and fitted the two rows to generate a centerline [35]; Gu et al. (2023) estimated lateral and heading states from binocular trunk localization [43]; Liu et al. (2024) extracted guidance lines from stable ridge geometry [23]; and Xu et al. (2025) combined U-Net segmentation with center-point extraction, interpolation, and route computation [29]. The sequence shows that geometry has not disappeared with deep learning; instead, it is increasingly the mechanism that converts learned semantics into stable control states. A representative route-calculation process is shown in Figure 4.

The sources of geometric evidence have broadened from image-derived boundaries to range sensing and metric row-state estimation. Once a reference is available, Hybrid A*, trajectory optimization, RRT-family methods, graph search, and potential-field planning can impose nonholonomic constraints, minimum turning radius, and obstacle clearance [63,64,65,66,67,68,69,70,71,72]. Table 4 emphasizes the operating conditions in which these geometric interfaces are useful and the failure modes that can propagate to control.

From an engineering perspective, the principal strength of geometric processing is interpretable error suppression and constraint enforcement. RANSAC can reject outliers, temporal filtering can reduce frame-to-frame reference jitter, and spline or curvature constraints can improve trajectory continuity. These operations are valuable only when the underlying observation remains physically meaningful; they should therefore be combined with confidence monitoring rather than used to cosmetically smooth a failed perception output.

Importantly, geometric regularization can stabilize observations that remain physically meaningful, but it cannot reliably reconstruct a road or row reference that has been completely lost or systematically misperceived. Future post-processing may therefore include explicit confidence, observability, and failure detection so that the system can distinguish random noise from front-end perception failure and enter a degraded or relocalization mode rather than continuing to output a visually smooth but physically incorrect reference.

### 3.2. Deep-Learning Detection and Segmentation: Robust Semantic Perception for Complex Orchard Scenes

Deep learning has substantially improved orchard visual perception under complex backgrounds and irregular appearance. Object detectors provide discrete targets such as trunks, people, and obstacles; semantic segmentation is suitable for roads, canopies, and free space; instance segmentation separates adjacent objects; and multi-task networks can share features between drivable-area and guidance-line prediction. For navigation, the important transition is that network outputs are increasingly designed to produce continuous geometric references and control-relevant variables rather than generic semantic labels. Representative learning-based studies are covered in Refs. [73,74,75,76,77,78,79,80,81]. Within this group, Wang et al. used ZED stereo vision with YOLOv4-Tiny to recover metric three-dimensional target positions [75], Zhao et al. combined depth vision with lightweight instance segmentation for low-cost metric plant-height estimation [76], and Sun et al. demonstrated transfer-learning-based organ detection under complex natural backgrounds [77]. Although these studies address different agricultural targets, they illustrate how learned semantic outputs can be extended toward metric localization and robust structural cues that are more directly usable by downstream navigation and control.

**Figure 5:**
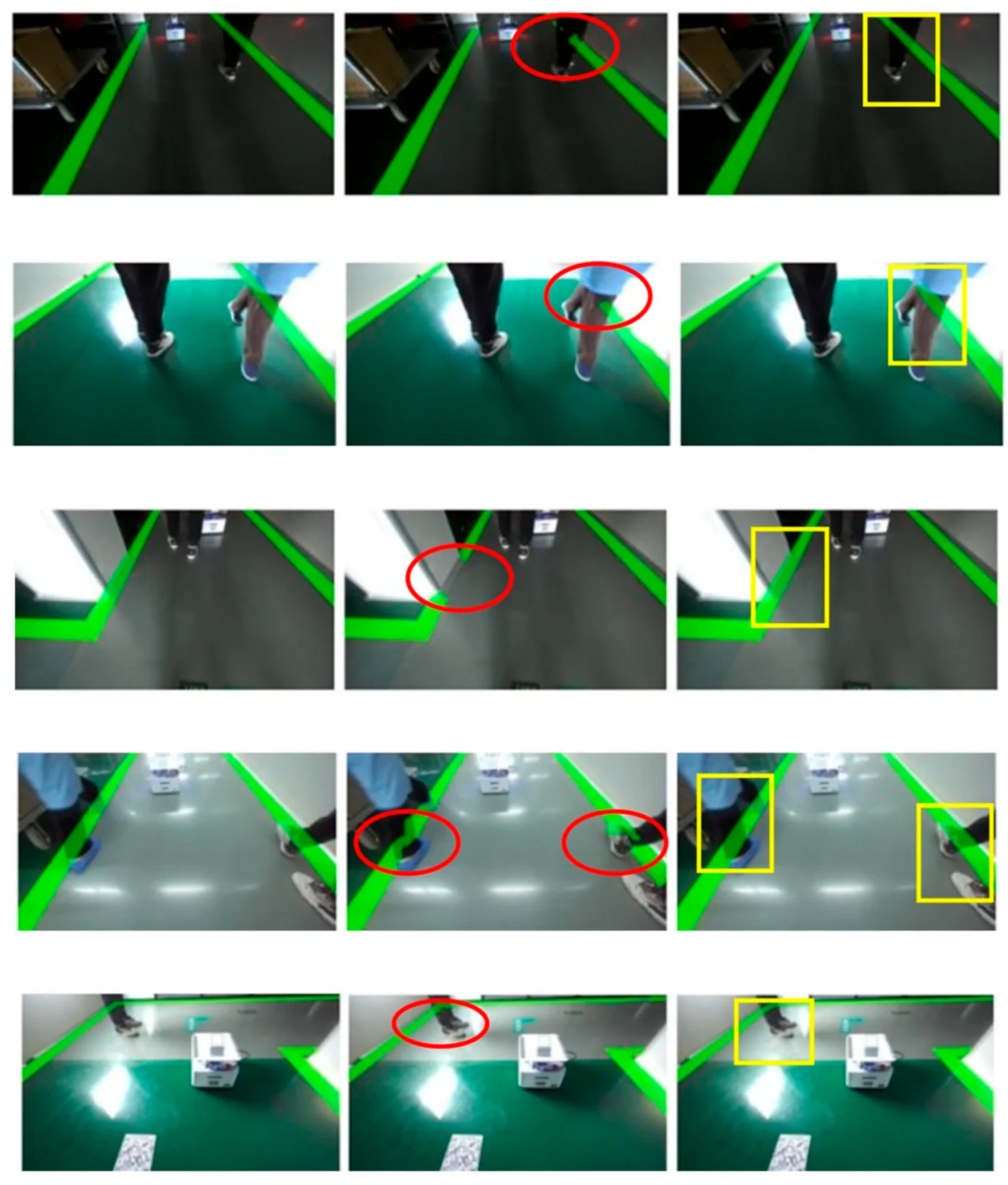
Lane-line detection robustness under multiple agricultural visual disturbances (reprinted from Ref. [27] under the Creative Commons Attribution 4.0 International license (CC BY 4.0)).

Representative work has progressed chronologically from lightweight line extraction to direct, disturbance-aware path generation. Zhang et al. (2024) proposed SN-CNN for lightweight seedling-guidance-line extraction [21]. In 2025, Lv et al. improved YOLOv8 for crop-free-ridge guidance-line recognition [22], Liu et al. developed a single-stage orchard navigation-path extraction network [78], and Wang et al. evaluated FSA-YOLOP under dim illumination, reflection, missing-line, and occlusion conditions [27]. These studies indicate that deep models are increasingly judged by whether they sustain a stable executable reference under navigation disturbances, not simply by image-level accuracy. Representative FSA-YOLOP results are shown in Figure 5.

Depth-aware learning extends the output from semantic masks toward metric scene constraints. RGB-D and binocular approaches attach range information to canopies, roads, and obstacles, while learning-based perception coupled with control becomes valuable when it reduces uncertainty and delay in the complete perception-to-control loop [79,80,81]. Table 5 compares these methods from an engineering rather than image-only perspective.

A further challenge is temporal behavior. Most training objectives remain frame based, whereas steering acts on a sequence of references; independent frame-wise errors can therefore become lateral jitter, curvature spikes, or short-lived safety-margin violations after geometric path construction. Possible control-oriented objectives include temporal centerline consistency, boundary continuity, confidence calibration, safety-critical false-negative penalties, and end-to-end latency.

A plausible next stage is a shift from image-metric-oriented deep learning toward control-oriented visual learning. In addition to mAP and mIoU, evaluation may report drivable-corridor stability, guidance-line bias and jitter, safety-critical miss rate, and end-to-end latency from image acquisition to control-reference generation. Linking these quantities to actual vehicle motion provides a more direct measure of engineering value.

### 3.3. Depth Vision, Visual SLAM and Multi-Sensor Fusion: Metric Localization Under Sensor Degradation

Monocular RGB vision provides rich semantics but limited absolute scale and short-term pose stability. Stereo and RGB-D cameras recover metric depth for trunks, obstacles, and road width; visual odometry and SLAM estimate local relative motion; and GNSS, IMUs, wheel odometry, AprilTags, and LiDAR provide complementary global, inertial, or geometric constraints. Canopy occlusion, repetitive rows, strong illumination, dynamic workers, and open headlands produce different degradation modes in orchards. Representative depth-vision, SLAM, and fusion studies are summarized in Refs. [82,83,84,85,86,87,88,89,90,91,92], with the primary engineering objective being continuous observability rather than simply a larger number of sensors.

**Figure 6:**
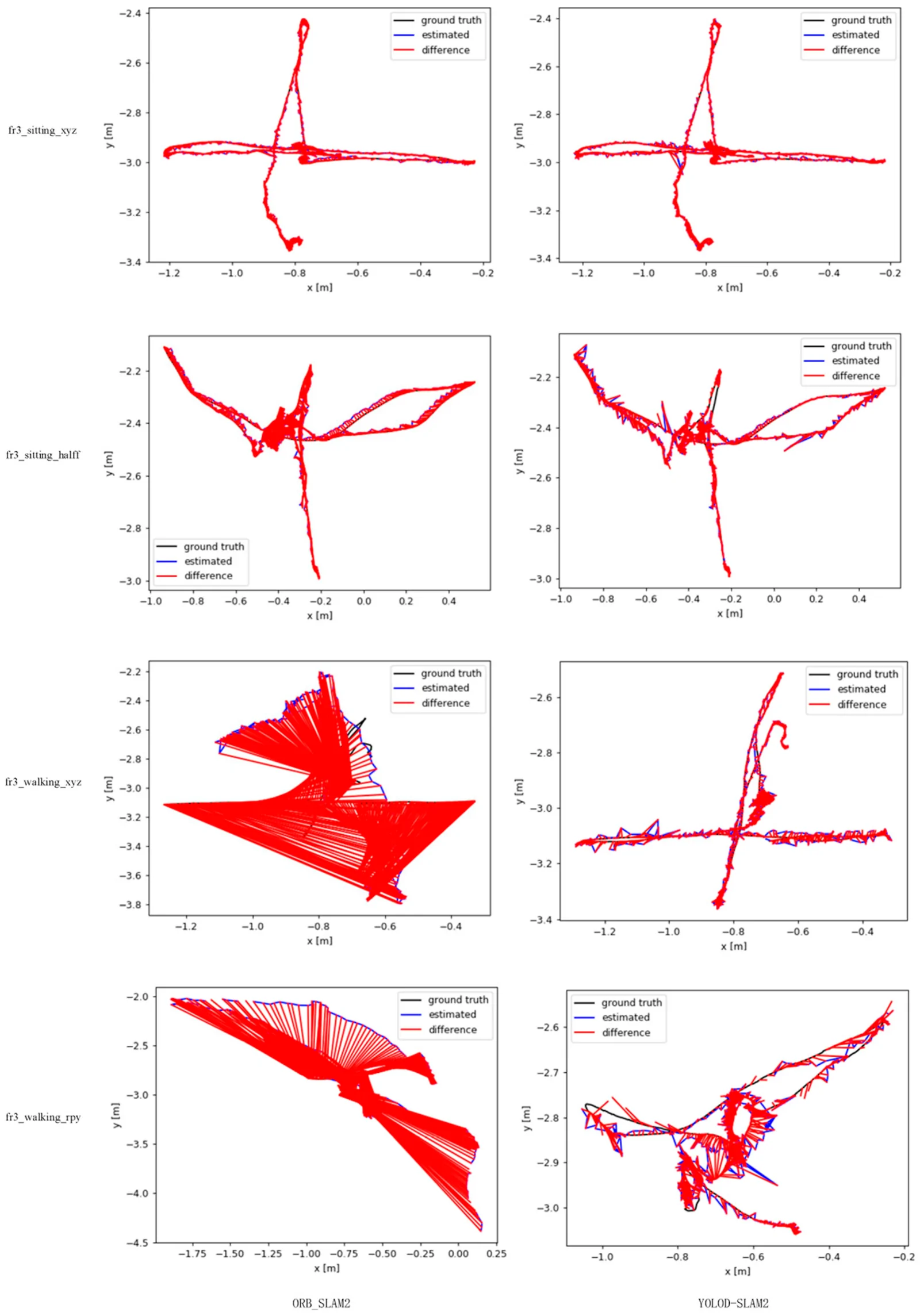
Trajectory comparison for visual SLAM localization in a dynamic orchard environment (reprinted from Ref. [85] under the Creative Commons Attribution 4.0 International license (CC BY 4.0)).

The chronology of orchard field systems shows a move from single-source localization toward degradation-aware multimodal navigation. Blok et al. (2019) demonstrated 2D-LiDAR probabilistic localization in a commercial apple orchard [40]. In 2024, Ma et al. suppressed dynamic visual interference in YOLOD-SLAM2 [85], Li et al. fused GNSS and LiDAR for intermittent satellite dropout [86], and Brown et al. combined RGB-D trunk semantics with particle filtering for in-row localization [87]. In 2026, Liu et al. explicitly switched between GNSS navigation outside rows and vision inside trellis rows [88], while Oh et al. validated camera, 2D LiDAR, IMU, and GNSS fusion together with low-speed path tracking for an orchard weeding robot [89]. This progression replaces permanent “all-sensor” fusion with scene-dependent use of the most trustworthy information. Representative YOLOD-SLAM2 results are shown in Figure 6.

Complementary approaches use artificial landmarks, point-cloud maps, LiDAR row centerlines, stereo visual odometry, and multi-sensor fusion to provide alternative geometric constraints when one modality becomes weak. Table 6 therefore compares not only sensor combinations but also the degradation mode each configuration is intended to tolerate. In practice, calibration, time synchronization, coordinate-frame management, and sensor-health estimation are part of the localization problem because their errors enter the controller in the same way as pose-estimation error.

Functionally, vision–IMU fusion primarily improves short-term pose continuity, GNSS–LiDAR fusion compensates for missing global position, visual SLAM emphasizes local map consistency, and tree-row structure provides a persistent scene prior. No single combination is universally optimal; robustness depends on whether the system can recognize the current degradation mode and adjust fusion weights or switch navigation modes before localization error propagates into steering control.

A possible next stage is uncertainty-aware fusion in which the controller receives not only a pose estimate but also covariance, sensor-health information, and scene-degradation indicators. These variables may trigger speed reduction, enlarged safety margins, re-anchoring of local drift with global information, or a controlled degraded state. Evaluation can therefore extend beyond mean localization error to loss-of-lock recovery time, mode-switch stability, and closed-loop navigation continuity.

LiDAR-based row-centerline extraction and stereo visual-odometry/SLAM studies further bridge metric localization with navigation-reference generation [93,94], providing the immediate interface to the planning and control methods discussed in Section 4.

## 4. From Visual Perception to Navigation Decisions and Motion Control

### 4.1. Navigation-Reference Construction and Control-Variable Extraction: Mapping Perception to Executable Geometry

The central interface problem in orchard visual navigation is how to convert scene observations into geometric and state variables that a vehicle can execute. Road masks, trunk detections, point clouds, and SLAM poses are not steering commands. They must be organized in the robot coordinate frame into a reference centerline, drivable corridor, or local target and then combined with vehicle kinematics to generate lateral error, heading error, reference curvature, look-ahead points, left/right clearance, reference speed, and validity/confidence flags [95,96,97]. For two detected corridor boundaries *b_L_*(*s*) and *b_R_*(*s*), the nominal centerline can be written as $c(s)=\frac{b_{L}(s)+b_{R}(s)}{2}$. For the first order, boundary biases *δb_L_* and *δb_R_* produce a centerline displacement $\delta c\approx \frac{\delta b_{L}+\delta b_{R}}{2}$, whereas their difference changes the estimated corridor width. Over a look-ahead distance *L*, a spatial change Δ*δ*_c_ produces an approximate heading perturbation $\delta \psi \approx \frac{\Delta \delta c}{L}$. Navigation-reference construction is therefore simultaneously the back end of perception and the front end of motion control.

**Figure 7:**
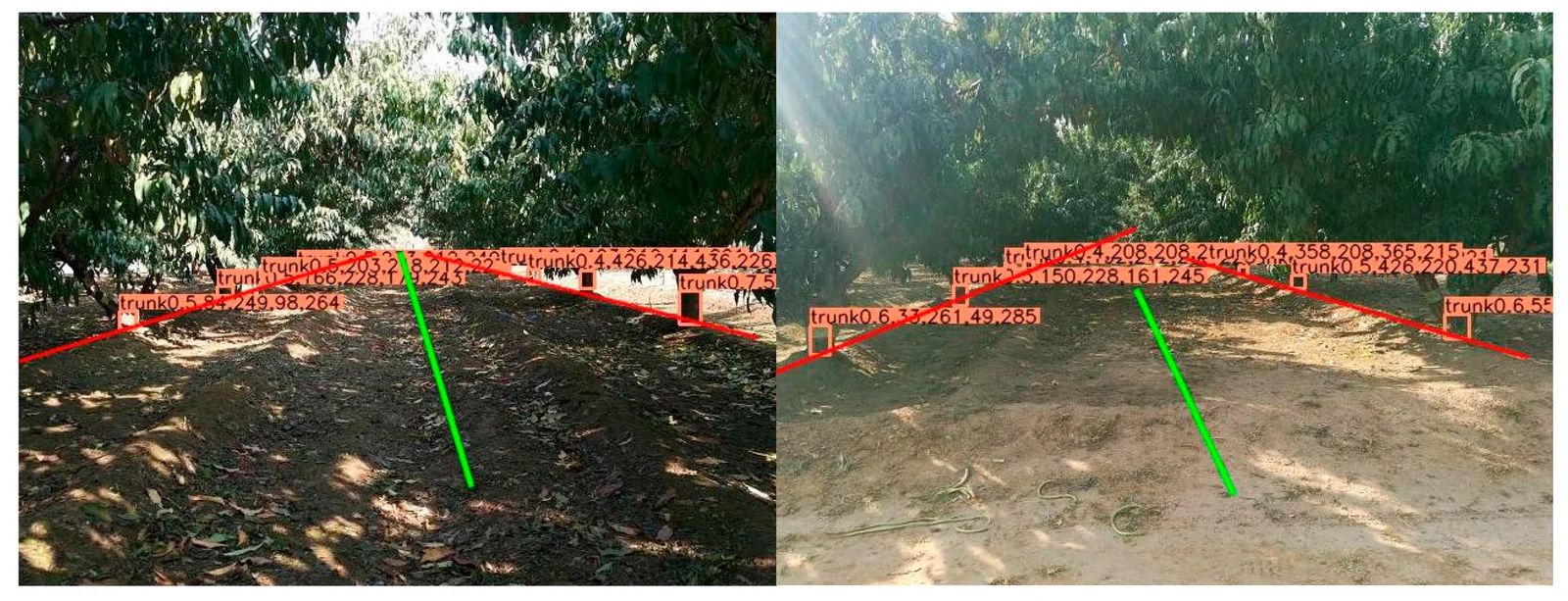
Centerline extraction from fruit-tree rows for orchard machinery navigation (reprinted from Ref. [35] under the Creative Commons Attribution 4.0 International license (CC BY 4.0)).

Representative studies show how this interface has expanded from local geometry to task-level paths. Zhou et al. fitted the two orchard rows from detected trees and generated a center guidance line, directly converting semantic landmarks into a steering reference [35]. Gu et al. further estimated robot position relative to fruit-tree rows so that image observations became lateral and heading states [43]. At the local-planning level, Li et al. combined laser SLAM with improved A* and DWA to generate trackable paths under obstacle constraints in dense orchards [98], while Xu et al. used UAV-derived tree positions and planting-row information for global orchard-vehicle route planning [99]. These studies reflect a transition from a single centerline toward hierarchical path representations that simultaneously encode local feasibility, vehicle constraints, and task structure. Representative orchard centerline extraction based on tree-row structure is shown in Figure 7.

Reference construction has expanded from local row centerlines to trajectory optimization, headland planning, coverage, and multi-row coordination [100,101,102,103,104,105,106,107,108,109,110,111,112,113,114,115,116,117,118,119,120,121,122,123]. Table 7 compares representative methods by the executable output they provide, the conditions for which they are suitable, and the failure mode that can propagate into control.

From a control perspective, geometric correctness does not guarantee executability. A collision-free path may still contain curvature discontinuities that induce steering oscillation, whereas a smooth path may pass too close to trunks when localization uncertainty is high. The interface should therefore communicate structured local states—reference geometry, clearance, validity, and confidence—rather than sparse waypoints alone.

A control-oriented navigation reference should therefore include path coordinates, local curvature, left/right clearance, validity, and confidence, while supporting the continuous transition from row following to headland turning, re-entry into the next row, and task continuation. Such a representation allows the controller to distinguish geometric error, perception degradation, and task-state change instead of attributing all deviations to tracking control.

At a minimum, the perception-to-control interface should provide the current look-ahead point or local reference segment, signed lateral error, heading error, local curvature and its rate of change, left/right clearance, reference speed, and validity/confidence flags. Representative implementations of these variables are summarized in Table 7.

An important research gap is independent evaluation of the mapping process itself. Reference-line bias, frame-to-frame jitter, geometric-processing delay, curvature distortion, and erroneous corridor contraction can enter the closed loop as disturbances even when the front-end detector remains statistically accurate. For a local controller linearized around the nominal path, the steering perturbation can be expressed generically as $\Delta u\approx K_{y}\Delta e_{y}+K_{\psi }\Delta e_{\psi }+K_{\kappa }\Delta \kappa$, where Δ*e_y_*, Δ*e_ψ_*, and Δ*κ* denote perception-induced lateral, heading, and curvature errors. End-to-end latency τ adds a stale-reference displacement of approximately *vτ* at vehicle speed v; on curved paths this also increases phase lag and can amplify overshoot or oscillation. Reporting perception metrics, reference-geometry quality, and control-input stability together therefore enables performance loss to be attributed to perception, mapping, latency, or control.

### 4.2. Vision-Guided Path Tracking and Motion Control: Converting Navigation References into Stable Vehicle Motion

Path tracking is the stage at which visual-perception error, reference-geometry error, and vehicle-dynamic constraints jointly become physical motion. Controller performance is therefore inseparable from perception quality: geometric controllers require stable lateral and heading errors, model-predictive control depends on sufficiently accurate states and dynamics, robust control focuses on external disturbances and parameter uncertainty, and learning-based control depends on coverage of relevant operating conditions. For orchard visual navigation, the controller should consequently be treated as one component of the perception–reference–execution chain rather than as an isolated downstream module [124,125,126,127,128].

**Figure 8:**
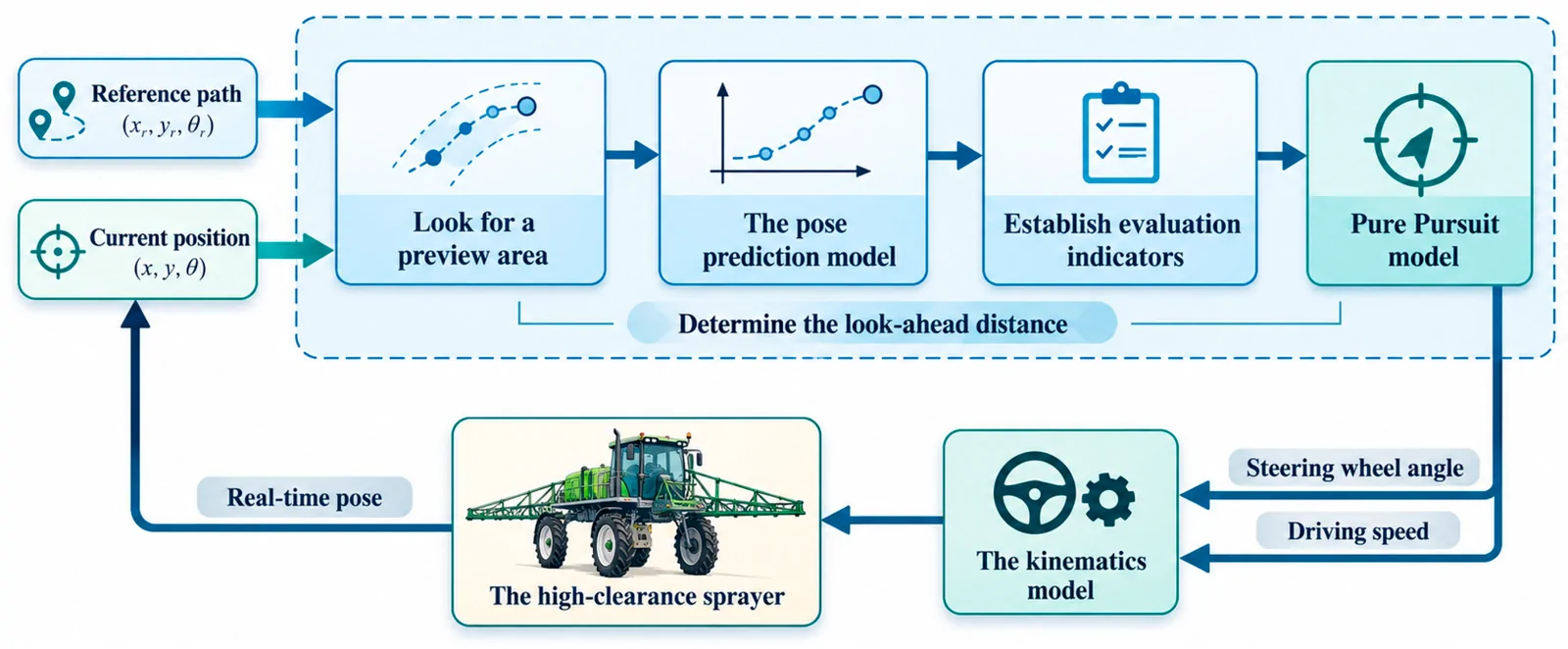
Closed-loop path-tracking framework with adaptive look-ahead distance (adapted from Ref. [132] under the Creative Commons Attribution 4.0 International license (CC BY 4.0)).

Representative controllers in this stage are covered in Refs. [129,130,131,132]. The control literature shows a chronological expansion of the stable operating envelope. Opiyo et al. (2021) coupled machine-vision path extraction with fuzzy steering control in an orchard [41]. Raikwar et al. (2022) used model-based navigation and control for a four-wheel-steered orchard robot under GNSS-denied operation [123], while Li et al. (2023) incorporated slip-aware cascaded MPC for orchard mowing [129]. In 2024, Cui et al. coordinated dynamic path search with fuzzy Stanley control [130] and Sun et al. introduced disturbance-observer-based fixed-time sliding-mode tracking [131]. In 2025, Wang et al. used a self-adjusting look-ahead distance for high-clearance sprayers [132]. Across these studies, the progression is from fixed geometric feedback toward model adaptation, constraint handling, disturbance compensation, and latency-aware preview. A representative adaptive look-ahead tracking framework is shown in Figure 8.

Subsequent research has strengthened the closed loop through predictive, adaptive, robust, and learning-assisted control [125,126,127,128,129,130,131,132,133,134,135,136,137,138,139,140,141,142,143,144,145,146,147,148,149,150,151,152,153,154,155,156,157,158,159,160]. Rather than implying a universally superior controller, Table 8 compares the operating condition each controller addresses, its principal engineering advantage, and the limitation that remains dependent on reference quality, model accuracy, terrain, or delay.

More recent work on nonlinear MPC, iterative learning, adaptive preview, and parameter optimization has increased the use of vehicle constraints and repetitive-operation characteristics [144,145,146,147,148]. Dynamic, slip-aware, and look-ahead studies for tracked platforms and complex terrain further improve representation of real execution behavior [149,150,152,156]. Efficiency-oriented MPC, disturbance-observer sliding-mode control, and reinforcement-learning tracking extend the objective to computational efficiency, disturbance recovery, and policy adaptation [139,154,155,158], while curvature-aware and learning-enhanced tracking further emphasize the dependence of control performance on reference geometry [153,157,159,160].

End-to-end latency is another indispensable closed-loop variable. Camera exposure, neural inference, geometric post-processing, communication, actuator response, and vehicle motion jointly produce a speed-dependent phase lag. State prediction, dynamic look-ahead, and speed scheduling can partially compensate for this delay only when reference quality and processing period are known online. Reporting network inference time alone is therefore insufficient to characterize real control-time performance.

Collectively, these studies indicate that the objective is not to identify a single universally optimal controller, but to establish a clear match between controller structure and reference quality, vehicle speed, curvature, slip, actuator limits, and delay. Because no controller can indefinitely compensate for a biased or discontinuous visual reference, robustness must be distributed across perception, reference construction, and vehicle control. Representative geometric, optimization-based, robust, and learning-based path-tracking studies for agricultural and orchard robots are summarized in Table 8.

A rigorous closed-loop evaluation can report mean, 95th-percentile, and maximum lateral error; heading error; overshoot and convergence distance; steering-rate smoothness; vehicle speed; end-to-end latency; number of human interventions; and success rate over complete multi-row operations. Image-level accuracy remains useful as an upstream diagnostic, but the system-level criterion is whether the complete perception–reference–control chain maintains stable, safe, and continuous motion under realistic orchard disturbances.

### 4.3. Task-Oriented Orchard Navigation: Coupling Mobility with Agricultural Execution

The engineering value of navigation is ultimately determined by the agricultural task, and different operations impose different tolerances on pose, speed, stopping behavior, and clearance. Spraying couples lateral position and canopy distance with nozzle timing; mowing and weeding require tight tree clearance; harvesting requires repeatable stopping within manipulator reach; transport prioritizes continuous row-to-row movement; and scouting requires repeatable sensing viewpoints. Because no universal numerical standard exists across platforms, Table 9 reports representative field values where the reviewed studies provide them and otherwise states the task-dependent requirement explicitly rather than imposing an unsupported threshold.

In practical implementation, however, the same perception–localization–planning–control chain is configured differently by task. For spraying, perception emphasizes canopy and row geometry, localization must maintain a smooth row-relative pose, and planning/control must synchronize vehicle speed, spray timing, and sprayer clearance. For harvesting, the chain shifts toward precise local pose and terminal stopping, with planning constrained by manipulator reachability and collision-free access. For mowing, near-trunk free-space and obstacle perception dominate, requiring rapid local replanning and conservative control margins around the cutting-tool envelope. For transport, localization continuity and route-level planning across rows and headlands are primary, while control emphasizes uninterrupted motion, safe row transitions, and reliable stopping at loading or unloading points. Thus, the framework is shared at the architectural level, but the perception outputs, localization precision, planning constraints, and control objectives are explicitly task dependent.

**Figure 9:**
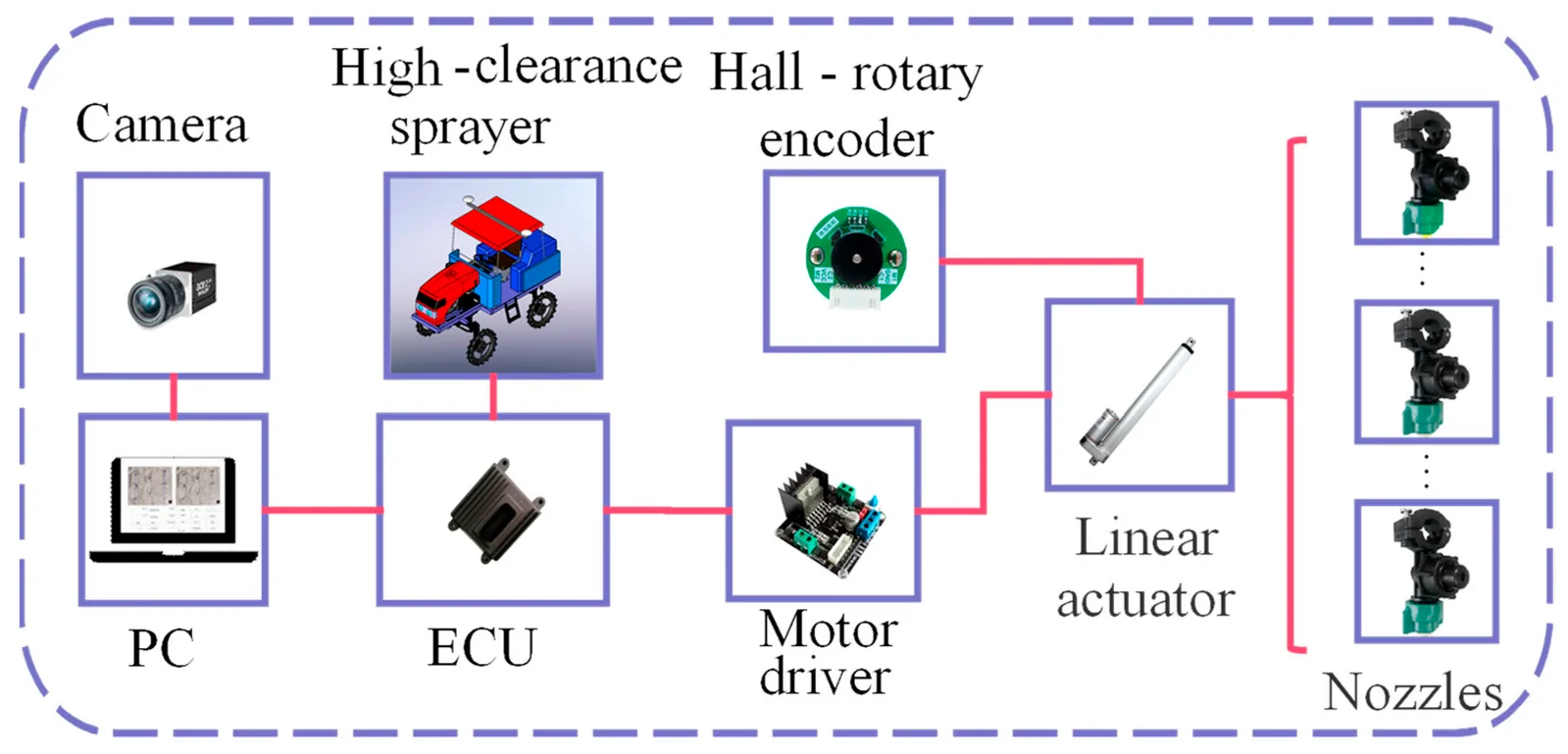
Transferable example of a vision-guided row-oriented spraying control architecture (reprinted from Ref. [161] under the Creative Commons Attribution 4.0 International license (CC BY 4.0)).

Representative systems already demonstrate explicit coupling between navigation and the agricultural actuator. Zheng et al. developed a machine-vision row-oriented spraying system that integrates visual guidance, vehicle speed, delay compensation, lateral actuation, and feedback in a unified control architecture [161]. Huang et al. designed an automatically guided fruit-harvesting platform, showing that harvesting directly tightens requirements on vehicle localization and stopping pose [162]. Jiang and Ahamed used trunk detection for autonomous orchard-sprayer navigation, coupling inter-row positioning with crop-protection operation [163], while Liu et al. developed an intelligent multivariable orchard spraying robot in which navigation state is coordinated with multi-variable plant-protection execution [164]. These systems demonstrate that operational constraints are not appended after navigation; they must be considered during path generation and control. The architecture of a representative machine-vision row-oriented spraying system is shown in Figure 9.

Task-specific studies make these constraints explicit. Manipulator and harvesting research shows that stopping pose and vehicle path must remain compatible with end-effector reachability and collision-free manipulation [165,166,167,168,169,170]. Pruning, fertilization, and spraying studies likewise redefine acceptable tree clearance, coverage geometry, and path order according to the operation [171,172,173,174,175]. Thus, a small navigation error can be operationally significant when it shifts the robot outside the workspace required by the downstream mechanism.

When operation expands to multiple rows or multiple robots, the objective shifts from local tracking accuracy toward task continuity and resource coordination. Coverage planning, multi-machine scheduling, and multi-UGV crop-protection studies show that row assignment, headland turning, conflict resolution, and recovery policy can dominate total work efficiency [176,177,178,179]. Global task state, local visual tracking, and actuator state should therefore share a consistent representation of row identity, progress, and resource availability.

Continuous autonomy also requires recovery from local failure and reliable task-state transition. Orchard spraying and mowing-planner studies indicate that low error on a single straight row is insufficient evidence of autonomous capability; the robot must also detect row ends, turn, relocalize in the next row, and resume the operation [174,180]. These system-level functions should become central evaluation items as orchard visual navigation moves from algorithm demonstrations toward deployable equipment. Representative systems are summarized in Table 9.

**Table 9:** Task-dependent navigation requirements and representative field values for orchard operations. Numerical values are reported examples, not universal standards.

Task	Lateral/Tracking Accuracy	Stopping-Position Requirement	Safety-Clearance Requirement	Operating Speed/Continuity	Localization Requirement
Spraying	Position RMSE of 0.114–0.155 m was reported across orchard/grass/concrete conditions in a LiDAR spraying prototype [163].	Usually secondary during continuous spraying; becomes important at row ends or task switching.	Must preserve canopy/tree clearance after accounting for vehicle width, spray-device envelope, and localization uncertainty.	Continuous low-speed row following; speed must remain synchronized with spray actuation.	Stable row-relative pose is required; local consistency can be more important than absolute global position under canopy.
Mowing/weeding	A field-validated multimodal orchard-weeding framework reported an average trajectory error of 0.1166 m [89].	Required for obstacle stops, turns, and safe restart; tolerance is platform specific.	Tight near-trunk clearance; the safety margin should include tool offset and pose uncertainty.	Low-speed continuous operation with reliable recovery after local avoidance and row transition.	The same framework reported a mean fused localization error of 0.0078 m [89]; synchronization remains critical.
Harvesting/pruning	An automatic fruit-picking platform reported maximum lateral deviations of 0.1015 m on straight paths and 0.1486 m on U-shaped paths [162].	High importance: the vehicle must stop within the manipulator reachable workspace; the exact tolerance is mechanism specific.	Vehicle and manipulator collision envelopes jointly define minimum clearance.	0.4 m/s was reported for the fruit-picking platform [162]; precise stop/restart is more important than high speed.	Decimeter-scale path tracking must be complemented by repeatable local stopping pose for manipulator access.
Transport	GNSS out-of-row average lateral deviation of 0.093–0.221 m and in-row visual heading deviation of about 5.23° were reported [88].	Moderate; mainly relevant at loading/unloading points and row-transition events.	Row-width dependent; clearance must be maintained through sensor-mode transitions.	0.6 m/s was reported in trellis-orchard tests [88]; continuous row-to-row operation is a primary objective.	Mode-dependent GNSS/vision localization must remain continuous across entry/exit transitions [88].
Scouting/inspection	No common numerical threshold is reported across the reviewed corpus; repeatable trajectory and viewpoint are the primary requirements.	Only required when station keeping or repeated viewpoint capture is part of the sensing protocol.	Must protect the sensor field of view and prevent branch/vehicle contact; value is platform specific.	Low-to-moderate speed is task dependent; repeatable revisit paths are often more important than throughput.	Relative pose repeatability and viewpoint consistency may be more important than absolute global precision.

Task-oriented navigation therefore expands the objective from minimizing tracking error to reliably completing the agricultural operation. Reducing speed may improve visual stability but lower field efficiency; the globally shortest route may be unsuitable for multi-robot coordination; and a lateral error acceptable for transport may be unsafe for near-trunk mowing. Navigation studies can therefore report task-specific tolerances together with task-completion rate, continuous-operation success, operation quality, and safety.

### 4.4. Integrated Perception–Localization–Planning–Control Chain and System Evaluation

The evidence reviewed above indicates that orchard visual navigation is most appropriately evaluated as a coupled perception–localization–planning–control–task-execution system. Error propagation can be traced explicitly. A segmentation bias changes the two corridor boundaries and therefore the centerline; centerline bias enters lateral and heading error; the controller converts these biased states into a steering command; and the resulting vehicle motion changes the camera viewpoint in the next frame. Localization drift produces an analogous path-registration error, whereas latency converts a correct but stale estimate into a speed-dependent spatial offset. Table 10 makes these propagation paths, observable symptoms, and mitigation variables explicit.

**Figure 10:**
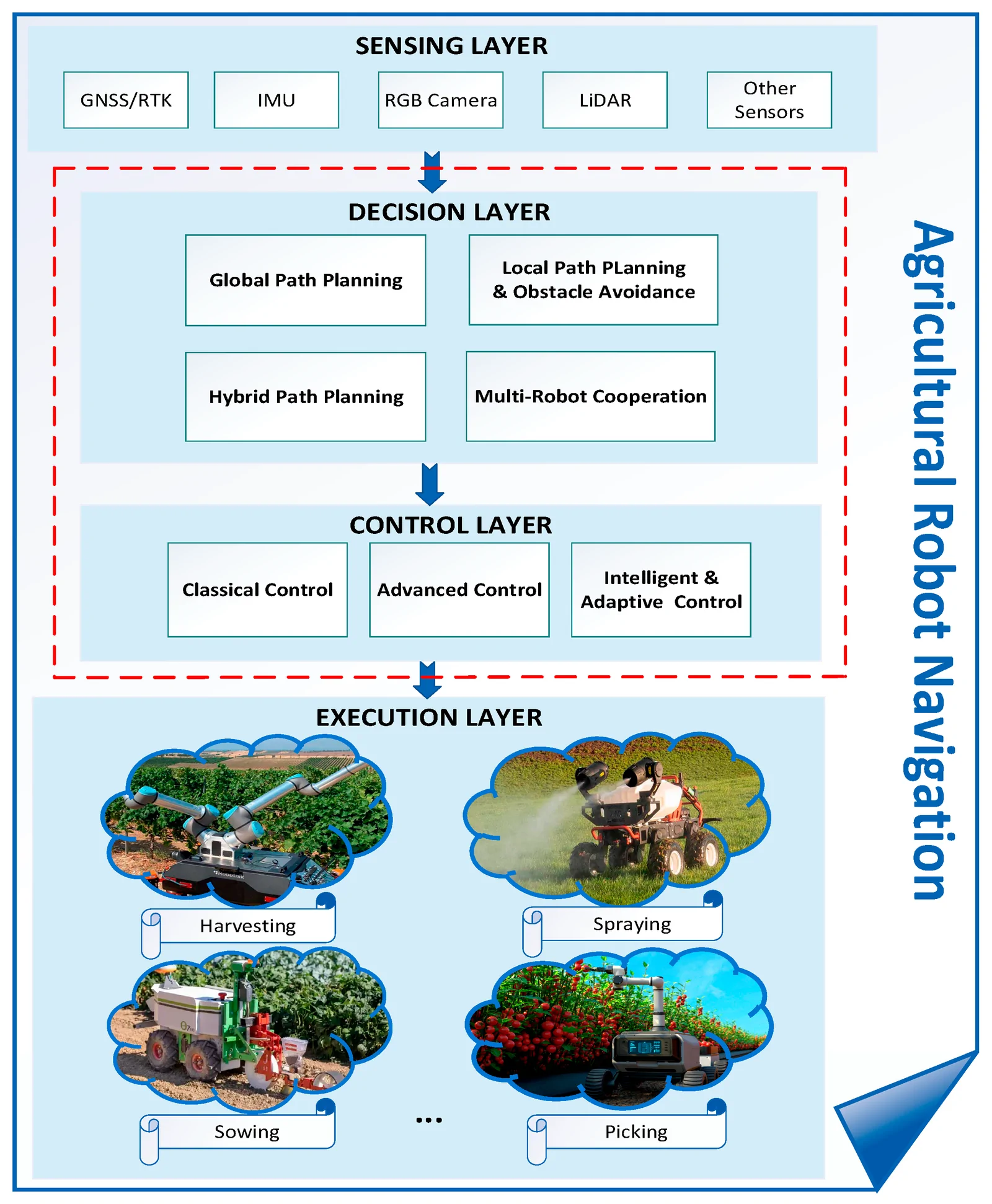
Reference architecture of an autonomous navigation system for agricultural robots (reprinted from Ref. [19] under the Creative Commons Attribution 4.0 International license (CC BY 4.0)).

System-level research is consequently moving from module integration toward interface consistency and fault recovery. The agricultural-robot navigation architecture proposed by Ye et al. places perception, decision making, control, and task execution within a unified technical stack and provides a structural basis for end-to-end evaluation [19]. Work in hilly orchards further demonstrates how upstream perception and localization errors can propagate through path references and controller inputs to the closed-loop vehicle response [20]. GNSS–vision mode switching in trellis orchards shows that the navigation source should change actively with observability [88], while autonomous spraying and multi-robot crop-protection systems require navigation state to remain consistent with operation execution, task progress, and scheduling [163,164,179]. A representative autonomous-navigation architecture for agricultural robots is shown in Figure 10.

System evaluation can synchronize perception outputs, estimated pose, reference paths, controller inputs, actuator feedback, and ground-truth trajectory on a common time base so that causal relationships among modules are preserved. Table 10 details representative error-propagation paths; Table 11 summarizes the mapping from information types to control variables and robot actions; and Table 12 translates the full-chain concept into a minimum benchmark protocol for orchard navigation.

Full-chain evaluation prevents strong local metrics from being misinterpreted as system reliability. A path-tracking controller may report centimeter-level error while the visual reference fails intermittently; a spraying robot may remain centered between rows without maintaining the canopy clearance required by the operation; and a multi-robot system may terminate a mission because of row-assignment or recovery failure despite accurate local trajectories. Comparable benchmarks can therefore report reference-line bias and jitter, localization drift, closed-loop tracking error, end-to-end latency, recovery time, manual-intervention frequency, row-transition success, and task-completion rate within the same experimental protocol.

Task-quality sensing provides a complementary evaluation perspective. In rice harvesting, Chen et al. combined a dedicated sampling device, controlled lateral illumination, and HSL color classification to estimate grain breakage rate; laboratory tests on approximately 2300 particles achieved 96% recognition accuracy [181]. This cross-domain example illustrates how visual classifications can be converted into an operation-specific quality indicator. For orchard robots, the corresponding methodological implication is to evaluate navigation together with independently measured task quality, such as spray coverage or mowing completeness. The harvesting study itself does not validate orchard navigation or autonomous feedback control.

## 5. Challenges and Future Directions

### 5.1. Current Challenges

The literature reviewed above indicates that the remaining challenges in orchard visual navigation are no longer confined to the accuracy of individual perception or control modules. Instead, they increasingly arise from the interaction among environmental variability, sensing degradation, reference construction, localization uncertainty, and closed-loop vehicle response. From a system-level perspective, the principal challenges can be grouped into the following aspects:

- Coupled environmental disturbances. Direct sunlight, backlighting, canopy shade, dust, vibration, wet ground, seasonal foliage variation, and repetitive tree textures can simultaneously degrade segmentation, landmark detection, and visual odometry. Canopy cover may also reduce GNSS availability at the same time, causing multiple sensing modalities to deteriorate concurrently rather than independently [20,48].
- Downstream sensitivity to perception errors. The same image-space error may have little practical effect in a wide alley but become safety-critical when the robot operates close to trunks or other obstacles. Its influence is further amplified by vehicle speed, steering delay, and path curvature. Therefore, control-relevant uncertainty is better characterized through reference-geometry error, state-estimation bias, and closed-loop response than through image-level accuracy alone [20].
- Generalization, computation, and field validation. Differences in tree species, training systems, pruning practices, seasonal canopy density, and ground management create substantial domain shift. At the same time, larger perception models introduce a latency–accuracy trade-off, while many studies still emphasize short straight-row trials rather than headland turning, failure recovery, and long-duration task execution [1,5,16]. Reported cases under low illumination, missing guidance lines, dynamic visual interference, and GNSS dropout further indicate that field disturbances often occur in combination rather than in isolation [27,45,85,86].
- Limited uncertainty representation. Many existing systems provide a path or pose estimate without a calibrated measure of confidence or reliability. This limits the ability of downstream modules to adjust speed, enlarge safety margins, reweight sensors, switch navigation modes, or initiate a controlled stop when sensing quality deteriorates.
- Incomplete full-chain evaluation. Reference bias, synchronization error, actuator delay, intervention frequency, recovery time, and task-completion quality are rarely reported within the same experimental framework. This limits engineering comparison among studies and makes it difficult to determine whether a performance loss originates from perception, localization, planning, control, or their interaction.

Among these failure modes, the most critical barriers to reliable deployment are those that cause a loss of observability or unsafe state transitions across multiple modules: prolonged canopy-induced GNSS degradation combined with visual or landmark loss, severe occlusion or missing row features that make the navigation reference temporarily unobservable, and delayed or inconsistent perception of dynamic obstacles. These compound failures are more consequential than isolated moderate accuracy losses because they can simultaneously corrupt localization, reference construction, and control. Priority should therefore be given to uncertainty-aware multimodal fusion with explicit sensor-health monitoring and graceful degradation; temporal structural perception and relocalization for missing or occluded landmarks; prediction-aware dynamic-obstacle tracking coupled directly to speed and clearance control; and long-duration field benchmarks that quantify recovery time, human intervention, and task completion after compound failures.

### 5.2. Future Development Directions

Building on these challenges, future research may increasingly focus on improving the robustness, interpretability, and system-level reliability of orchard navigation rather than pursuing isolated gains in individual algorithmic metrics. Several directions appear particularly relevant:

- Uncertainty-aware multimodal perception. Future systems may estimate the reliability of RGB, depth, LiDAR, GNSS, and inertial observations online and propagate this information to downstream modules. Confidence-aware strategies could dynamically reweight sensing sources, reduce vehicle speed, enlarge safety margins, retain the last reliable reference for a bounded interval, or trigger a controlled stop when perception reliability falls below an acceptable level.
- Control-oriented perception. Perception models may be designed not only to optimize pixel-level accuracy, but also to reduce centerline displacement, corridor discontinuity, safety-critical obstacle misses, and reference-generation latency. In this context, a more meaningful validation criterion would be whether perception improvements reduce downstream reference bias, tracking error, or hazardous control responses rather than merely increasing mAP or mIoU.
- Edge deployment and virtual–real validation. Edge-computing and virtual–real workflows may be evaluated using end-to-end control latency, energy consumption, and their ability to reproduce field-relevant failure boundaries. When calibrated against real sensor response, tire–soil interaction, and actuator dynamics, digital twins could provide controlled testing of glare, sensor dropout, atypical obstacles, terrain variation, and slope-induced motion before field deployment.
- Intervention-aware reliability assessment. Future evaluation may include not only average tracking or localization accuracy, but also degradation-detection time, recovery time, row-transition success, task-completion rate, and the distance or duration of operation without human intervention. These indicators better reflect the practical autonomy of orchard robots over extended missions.
- Standardized full-chain benchmarks. A useful benchmark framework may synchronize images, depth or point-cloud data, sensor-health indicators, vehicle pose, reference paths, controller commands, actuator feedback, and task outcomes across different orchards, seasons, and operating conditions. Foundation or multimodal models may be particularly valuable when they address a clearly defined system bottleneck and remain separated from direct steering actuation through deterministic geometric representations, uncertainty checks, and explicit safety constraints.

## 6. Conclusions

Orchard visual navigation has progressed from color-threshold processing and single navigation-line extraction toward semantic scene understanding, three-dimensional localization, sensor fusion, and closed-loop motion control. The central technical transition is that perception outputs are increasingly required to carry explicit control meaning: a road mask must become a drivable corridor, trunk detections a row model, obstacle detections safety constraints, and pose estimates state feedback. This review therefore organizes the literature according to the chain from scene information to execution rather than by algorithms alone.

Three conclusions emerge. First, passable ground, tree-row structure, and task semantics are complementary navigation cues, and robust systems can switch or fuse them according to observation reliability. Second, deep learning is most effective when coupled with geometry, temporal filtering, and metric localization because controllers require stable coordinates and reference variables rather than isolated recognition outputs. Third, field performance is determined by the complete perception–localization–planning–control chain: reference bias, latency, vehicle dynamics, and task constraints can dominate closed-loop behavior even when perception accuracy is high.

Accordingly, a practical next generation of orchard robots may be organized around uncertainty-aware interfaces and full-chain validation. Progress can be assessed through continuous row-to-row autonomy, safe degraded-mode operation, task-completion quality, and long-duration robustness across orchards and seasons. The decisive criterion is not isolated visual accuracy, but whether scene information is converted into temporally stable, uncertainty-qualified, and dynamically feasible control variables throughout the agricultural mission.

## Figures and Tables

**Table 1:** Engineering comparison of representative passable-area and road-boundary methods, including operating conditions and dominant failure modes.

Researcher (Year)	Method	Control-Relevant Output	Operating Condition/Suitable Use	Engineering Strength	Dominant Limitation/Failure Mode	Ref.
Chen et al. (2021)	Dynamic ROI boundary measurement	Metric lateral distance	Visible harvesting boundary; low-compute guidance	Direct conversion from pixels to physical offset	Depends on stable boundary visibility and camera calibration	[24]
Liu et al. (2024)	Machine-vision ridge guidance	Guidance line/steering reference	Crop-free ridges with discernible geometry	Interpretable and computationally light	Sensitive to weak or occluded geometric features	[23]
Zhong et al. (2024)	Improved U-Net	Drivable-area mask	Low-light agricultural paths	Improves path extraction under illumination degradation	Mask quality can still fluctuate under extreme glare or occlusion	[26]
Wang et al. (2025)	FSA-YOLOP multi-task perception	Guidance-line geometry	Dim light, reflection, missing lines, occlusion	Explicit robustness evaluation across visual disturbances	Residual line loss can generate discontinuous references	[27]
Xu et al. (2025)	U-Net + center extraction/interpolation	Continuous navigation route	Orchard road with segmentable traversable region	Converts segmentation into trackable geometry	Interpolation cannot correct systematic segmentation bias	[29]
Zhang et al. (2026)	Improved YOLOv5s + multi-factor fusion	Passable region/safety state	Mixed agricultural road surfaces	Extends perception from boundary to traversability	Generalization depends on surface distribution and fusion thresholds	[30]

**Table 2:** Engineering comparison of trunk-, tree-row-, and canopy-based navigation landmarks.

Researcher (Year)	Sensing/Method	Control-Relevant Output	Operating Condition/Suitable Use	Engineering Strength	Dominant Limitation/Failure Mode	Ref.
Radcliffe et al. (2018)	Canopy–sky machine vision	Row-center steering cue	Peach orchard with visible canopy–sky boundary	Low-cost direct visual guidance	Requires persistent canopy–sky contrast	[39]
Blok et al. (2019)	2D LiDAR + PF/KF localization	Relative pose/lateral deviation	Commercial apple orchard; missing-tree patterns	Probabilistic localization tolerates partial row gaps	Depends on row-structure observability and scan geometry	[40]
Opiyo et al. (2021)	Machine vision + medial axis + fuzzy control	Guidance path and steering error	Orchard inter-row navigation	Direct path extraction coupled to control	Appearance/texture changes can degrade segmentation	[41]
Huang et al. (2023)	Trunk perception + depth + DWA	Metric trunks/local path	Orchard with detectable trunks	Links semantic landmarks to local planning	Occlusion and depth error reduce valid landmarks	[42]
Gu et al. (2023)	Binocular trunk localization + row fitting	Lateral and heading states	Fruit-tree rows with bilateral structure	Direct control-state estimation	Asymmetric landmark loss biases the fitted row axis	[43]
Zheng et al. (2026)	Target-oriented trunk detector	Trunk landmarks + confidence	Sunny, cloudy, shaded, missed detections	Explicit illumination-robust validation	Severe occlusion or missed trunks reduce structural support	[45]

**Table 3:** Engineering comparison of obstacle perception, action-relevant semantics, and local avoidance.

Researcher (Year)	Method	Decision Variable	Operating Condition/Suitable Use	Engineering Strength	Dominant Limitation/Failure Mode	Ref.
Inoue et al. (2022)	Machine vision + obstacle avoidance	Obstacle/free-space constraint	Orchard mowing near trees	Demonstrates integrated avoidance in narrow clearance	Avoidance may conflict with the primary row-following reference	[50]
Zhuang & Li (2023)	Segmentation + angle estimation	Region/angle state	Geometry-dominated crop scenes	Adds orientation information to semantic regions	Not designed for dynamic orchard obstacles	[59]
Chatzisavvas et al. (2024)	A*-based navigation	Collision-free path	Structured obstacle field	Simple global obstacle-aware planning	Static-map assumption limits response to moving obstacles	[60]
Syed et al. (2025)	CNN real/fake obstacle classification	Avoidance priority/semantic risk	Orchard clutter with vegetation and rigid objects	Links visual class directly to motion relevance	Misclassification can cause missed avoidance or false stops	[48]
Navone et al. (2025)	Depth semantic segmentation	Free-space corridor	GPS-free dense tree rows	Couples semantic free space with row following	Depth/segmentation degradation can shift corridor	[51]
Wang et al. (2025)	Improved dynamic-window approach	Velocity/local trajectory	Dynamic obstacles	Transforms obstacle observations into feasible motion	Late detection or stale velocity estimates reduce clearance	[52]

**Table 4:** Engineering comparison of classical vision and geometric processing for navigation-reference extraction.

Researcher (Year)	Method	Control Interface	Operating Condition/Suitable Use	Strength	Limitation/Failure Mode	Ref.
Radcliffe et al. (2018)	Canopy–sky feature geometry	Row-center cue	Orchard rows with visible sky gap	Very low computational burden	Strong dependence on canopy–sky visibility	[39]
Opiyo et al. (2021)	Gabor/PCA + medial axis	Trackable center path	Inter-row orchard navigation	Direct geometry-to-control pipeline	Texture/illumination shift can alter segmentation	[41]
Gu et al. (2023)	Binocular trunk geometry + least squares	Lateral/heading error	Bilateral tree rows	Physically interpretable state estimates	Outliers or asymmetric row visibility bias the fit	[43]
Fang et al. (2025)	DBSCAN-MFI clustering	Road-region geometry	Weak explicit road edges	Works without a crisp line boundary	Clustering parameters are scene dependent	[28]
Chi et al. (2024)	Improved Hybrid A*	Kinematically feasible reference path	Nonholonomic mobile robot	Explicit turning/kinematic feasibility	Requires map/obstacle accuracy and search tuning	[66]
Xu et al. (2024)	Improved A*	Global geometric path	Mapped mobile-robot environment	Simple and interpretable global planning	Static-map assumptions limit dynamic response	[67]

**Table 5:** Engineering comparison of deep-learning methods for control-oriented agricultural and orchard navigation.

Researcher (Year)	Network/Sensing	Control-Relevant Output	Operating Condition/Suitable Use	Strength	Limitation/Failure Mode	Ref.
Zhong et al. (2024)	Improved U-Net	Robust path mask	Low-light agricultural path	Improved illumination tolerance	Frame-wise masks may still jitter temporally	[26]
Zhu et al. (2024)	Improved DeepLabV3+	Road segmentation reference	Pitaya orchard with complex interference	Orchard-specific semantic robustness	Requires geometric post-processing for executable path	[31]
Wang et al. (2025)	FSA-YOLOP	Guidance-line geometry	Dim light, reflection, missing lines, occlusion	Multi-disturbance robustness evaluation	Severe line loss can create reference discontinuity	[27]
Liu et al. (2025)	Single-stage path extraction network	Direct navigation path	Orchard scenes requiring short processing chain	Reduces perception-to-path stages	Domain shift and temporal consistency remain concerns	[78]
Zhang et al. (2026)	Deep binocular vision	Metric scene/path geometry	Orchard robot with stereo visibility	Adds metric scale to learned perception	Stereo quality degrades with texture, occlusion, or calibration error	[81]

**Table 6:** Engineering comparison of depth-vision, visual-SLAM, and multi-sensor localization methods under sensor degradation.

Researcher (Year)	Sensor/Fusion Configuration	Localization/Control Output	Operating Condition	Strength	Dominant Degradation/Limitation	Ref.
Blok et al. (2019)	2D LiDAR + PF/KF	Relative pose/lateral deviation	Commercial apple orchard	Robust probabilistic in-row localization	Missing/nonuniform row structure affects localization	[40]
Ma et al. (2024)	Dynamic-object-aware visual SLAM	Visual pose	Dynamic orchard scenes	Suppresses moving-object interference	Repetitive structure and weak texture can still cause drift	[85]
Li et al. (2024)	GNSS + LiDAR	Continuous pose	Intermittent GNSS dropout	Maintains global/local continuity	Requires calibrated switching/fusion and reliable LiDAR geometry	[86]
Brown et al. (2024)	RGB-D trunks + particle filter	Tree-level in-row pose	Degraded GNSS	Uses persistent orchard landmarks	Trunk occlusion reduces measurement support	[87]
Liu et al. (2026)	GNSS–vision mode switching	Mode-dependent pose/reference	Trellis orchard inside/outside rows	Explicit observability-driven source switching	Transition discontinuity must be controlled at row entry/exit	[88]
Oh et al. (2026)	GNSS + IMU + 2D LiDAR + camera + EKF	Pose + low-speed path tracking	Structured orchard weeding	Field-validated multimodal navigation/control	Requires reliable synchronization and task/speed-specific tuning	[89]

**Table 7:** Engineering comparison of methods that convert perception or map information into executable navigation references.

Researcher (Year)	Input	Reference Construction	Executable Output	Suitable Condition/Strength	Limitation/Failure Mode	Ref.
Li et al. (2022)	Laser-SLAM orchard map	Improved A* + DWA	Global/local feasible path	Dense orchard with mapped obstacles	Map error or stale obstacles propagate into local path	[98]
Zhang et al. (2023)	Local obstacle geometry	Elliptic-repulsion potential field	Locally safe mower path	Reactive local avoidance	Potential-field local minima/tuning sensitivity	[114]
Wang et al. (2024)	Orchard-row environment	Improved A* + SVR	Trackable orchard-row path	Combines global search with smooth fitting	Path quality depends on map/reference accuracy	[118]
Ye et al. (2024)	Unstructured orchard map	Continuous bidirectional Quick-RRT*	Smooth feasible path	Complex nonholonomic spaces	Sampling variability and computation increase in clutter	[121]
Chen et al. (2025)	LiDAR row points	Multipoint centerline extraction	Orchard row centerline	Metric row geometry	Sparse/noisy trunk points distort centerline	[93]
Xu et al. (2025)	UAV tree positions + planting rows	Global route planning	Field-scale orchard route	Adds global row structure	Aerial-map registration error can shift ground execution	[99]

**Table 8:** Engineering comparison of representative path-tracking and motion-control methods.

Researcher (Year)	Controller	Primary Operating Condition	Strength	Dominant Limitation/Failure Mode	Ref.
Liu et al. (2021)	Fuzzy adaptive MPC	High-clearance sprayer; variable operation	Handles constraints and changing operating state	Model/optimization burden and state-estimation quality	[137]
Raikwar et al. (2022)	Model-based navigation/control	Four-wheel-steered orchard robot; GNSS denied	Explicit vehicle-model integration	Model mismatch and sensing limitations	[123]
Xu et al. (2022)	Efficiency-oriented MPC	Autonomous agricultural machinery	Balances tracking with computation	Requires accurate model and bounded latency	[139]
Lu et al. (2023)	Robust trajectory tracking	Tractor–trailer with uncertainty/disturbance	Improves tolerance to parameter uncertainty	Conservatism/tuning under changing terrain	[151]
Zhou et al. (2023)	Modified MPC	Articulated steering tractor	Accounts for articulated dynamics	State/model errors can degrade prediction	[153]
Sun et al. (2024)	Disturbance-observer fixed-time SMC	Agricultural vehicle under disturbances	Fast robust disturbance rejection	Chattering/actuator bandwidth and noisy state estimates	[131]
Wang et al. (2025)	Dynamic look-ahead tracking	Crawler orchard sprayer	Adapts preview to vehicle state	Poor reference quality cannot be compensated indefinitely	[156]
Zhang et al. (2025)	Curvature-aware reinforcement learning	Agricultural vehicle on varying curvature	Adaptive nonlinear policy	Generalization/safety assurance outside training distribution	[158]

**Table 10:** Representative error-propagation paths from perception/localization to closed-loop motion.

Upstream Error	Propagation Path	Control Manifestation	Field Consequence	Diagnostic Metric/Mitigation Variable
Boundary/segmentation bias	Boundary shift → centerline bias → *e_y_*/*e_ψ_* bias	Persistent steering offset	Systematic row offset or reduced tree clearance	Metric boundary bias; centerline RMSE; confidence-weighted fitting
Asymmetric landmark dropout	One-sided landmark loss → row-axis rotation → heading bias	Alternating or biased steering correction	Cross-row drift/oscillation	Valid-landmark count; left/right support ratio; robust-fit residual
Localization drift	Pose drift → path misregistration → incorrect *e_y_*/*e_ψ_*	Controller tracks a displaced path	Systematic tracking error despite smooth control	Absolute/relative pose drift; covariance; relocalization trigger
Perception/control latency τ	Stale pose/reference → spatial lag ≈ *vτ* → phase lag	Overshoot or oscillation at higher speed/curvature	Clearance loss and unstable tracking	End-to-end latency; time synchronization; speed scheduling/prediction
Obstacle-detection delay	Late obstacle update → underestimated occupancy/clearance	Delayed braking or avoidance	Minimum-clearance violation/emergency stop	Time-to-detection; TTC margin; obstacle-track age
Mode-switch discontinuity	Sensor/source switch → pose/reference jump	Steering impulse/transient command spike	Headland re-entry overshoot or loss of row	Reference jump magnitude; switch settling time; hysteresis/blending

**Table 11:** Integrated mapping from orchard scene information to control variables, robot actions, and evaluation metrics.

Scene/State Information	Enabling Technology	Control Variable Generated	Planning/Decision Use	Robot Action	Minimum Evaluation Metric
Passable area/road boundaries	RGB/depth segmentation + geometry	Centerline, left/right boundary, clearance, *e_y_*, *e_ψ_*, confidence	Corridor selection, speed and clearance constraints	Steering + speed regulation	Boundary/centerline bias, jitter, lateral/heading error
Trunks/tree rows/canopy	Detection/segmentation + depth/LiDAR + row fitting	Row axis, relative pose, landmark count, covariance	Pose correction, reference anchoring, confidence weighting	Row following/degraded-mode switch	Landmark stability, pose error, loss-of-lock/recovery
Obstacles/row ends/task events	Detection/segmentation + tracking	Obstacle extent/velocity, risk, row-end event	Local avoidance, stop/turn/re-entry state transition	Velocity change, avoidance, headland turn	Minimum clearance, event accuracy, row-transition success
Global/local pose and task state	VO/SLAM/GNSS/IMU/LiDAR + task manager	Pose, covariance, row ID, progress, actuator state	Path registration, replanning, task scheduling	Tracking command + actuator coordination	Pose drift, latency, intervention count, task completion

**Table 12:** Proposed minimum full-chain benchmark protocol for orchard visual navigation.

Scenario	Required Disturbance/Maneuver	Primary Metrics	Recovery/Task Metrics	Recommended Reporting Condition
Straight-row tracking	Nominal row at ≥2 speeds	Mean/95th/max lateral error; heading error; steering smoothness; latency	Interventions; traversal success	≥5 runs; report row width, speed, illumination, and surface
Row-end turning and re-entry	Leave row → headland turn → reacquire next row	Turn-path error; peak heading error; reference continuity	Row-transition success; settling distance/time	≥5 transitions in both turn directions
GNSS degradation/loss	Controlled GNSS dropout or canopy-loss segment	Pose drift; mode-switch delay; tracking error	Recovery time; continuity; intervention count	Report dropout duration and available sensors
Temporary visual occlusion	Occlude row/landmarks for a bounded interval	Reference age; centerline/pose drift; control latency	Time to stable reacquisition; safety-margin violation	Repeat across ≥2 occlusion durations
Dynamic obstacle appearance	Obstacle enters drivable corridor	Detection/track delay; minimum clearance; speed response	Avoidance success; false stop; task-resumption time	Report obstacle speed, size, and entry location
Localization failure/recovery	Forced pose loss or relocalization event	Pose jump; reference jump; controller transient	Recovery time; successful relocalization; intervention count	Report failure trigger and recovery mechanism
Continuous task mission	Multi-row spraying/mowing/transport/scouting sequence	Tracking error + end-to-end latency across mission	Row-transition success; task-completion rate; intervention-free distance/time	Report mission duration, rows, turns, and task-quality metric

## Data Availability

No new data were created or analyzed in this study. Data sharing is not applicable to this article.

## References

[1] Attri I., Awasthi L.K., Sharma T.P., Rathee P. (2023). A review of deep learning techniques used in agriculture. Ecol. Inform..

[2] Bai Y., Zhang B., Xu N., Zhou J., Shi J., Diao Z. (2023). Vision-based navigation and guidance for agricultural autonomous vehicles and robots: A review. Comput. Electron. Agric..

[3] Chakraborty S., Elangovan D., Govindarajan P.L., Elnaggar M.F., Alrashed M.M., Kamel S. (2022). A comprehensive review of path planning for agricultural ground robots. Sustainability.

[4] He Y., Huang Z., Yang N., Li X., Wang Y., Feng X. (2024). Research Progress and Prospects of Key Navigation Technologies for Facility Agricultural Robots. Smart Agric..

[5] Hossen M.I., Awrangjeb M., Pan S., Mamun A.A. (2025). Transfer learning in agriculture: A review. Artif. Intell. Rev..

[6] Jiang L., Xu B., Husnain N., Wang Q. (2025). Overview of Agricultural Machinery Automation Technology for Sustainable Agriculture. Agronomy.

[7] Jin Y., Liu J., Xu Z., Yuan S., Li P., Wang J. (2021). Development status and trend of agricultural robot technology. Int. J. Agric. Biol. Eng..

[8] Liu L., Wang X., Yang X., Liu H., Li J., Wang P. (2023). Path planning techniques for mobile robots: Review and prospect. Expert Syst. Appl..

[9] Mhamed M., Zhang Z., Yu J., Li Y., Zhang M. (2024). Advances in apple’s automated orchard equipment: A comprehensive research. Comput. Electron. Agric..

[10] Stano P., Montanaro U., Tavernini D., Tufo M., Fiengo G., Novella L., Sorniotti A. (2023). Model predictive path tracking control for automated road vehicles: A review. Annu. Rev. Control.

[11] Dolgov D., Thrun S., Montemerlo M., Diebel J. (2010). Path planning for autonomous vehicles in unknown semi-structured environments. Int. J. Robot. Res..

[12] Wang Q., He J., Lu C., Wang C., Lin H., Yang H., Li H., Wu Z. (2023). Modelling and control methods in path tracking control for autonomous agricultural vehicles: A review of state of the art and challenges. Appl. Sci..

[13] Wu H., Wang X., Chen X., Zhang Y., Zhang Y. (2025). Review on Key Technologies for Autonomous Navigation in Field Agricultural Machinery. Agriculture.

[14] Yao Z., Zhao C., Zhang T. (2024). Agricultural machinery automatic navigation technology. iScience.

[15] Moysiadis V., Tsolakis N., Katikaridis D., Sørensen C.G., Pearson S., Bochtis D. (2020). Mobile Robotics in Agricultural Operations: A Narrative Review on Planning Aspects. Appl. Sci..

[16] Ait Hammou N., El Aissaoui A., Abouch Y., Mousannif H. (2026). Vision-Based Crop Row Detection for Autonomous Agricultural Navigation: A Systematic Review and Practical Perspective of Developing Cost-Effective Field Robots. AgriEngineering.

[17] Qu J., Zhang Z., Qin Z., Guo K., Li D. (2024). Applications of Autonomous Navigation Technologies for Unmanned Agricultural Tractors: A Review. Machines.

[18] Boros N., Ambrus B., Nyéki A. (2026). Navigation and Sensor Fusion for Autonomous Field Robots in Precision Agriculture: Narrative Review. Sensors.

[19] Ye F., Le F., Cui L., Han S., Gao J., Qu J., Xue X. (2026). Application of Navigation Path Planning and Trajectory Tracking Control Methods for Agricultural Robots. Agriculture.

[20] Zhang Z., Wang Y., Lei H., Dong X., Jia W. (2026). Perception and Localization Error Propagation and Robust Path-Tracking Control for Agricultural Machinery in Hilly Orchards: A Review. Sensors.

[21] Zhang T., Zhou J., Liu W., Yue R., Shi J., Zhou C., Hu J. (2024). SN-CNN: A lightweight and accurate line extraction algorithm for seedling navigation in ridge-planted vegetables. Agriculture.

[22] Lv R., Hu J., Zhang T., Chen X., Liu W. (2025). Crop-free-ridge navigation line recognition based on the lightweight structure improvement of YOLOv8. Agriculture.

[23] Liu W., Hu J.P., Liu J.X., Yue R.C., Zhang T.F., Yao M.J., Li J. (2024). Method for the navigation line recognition of the ridge without crops via machine vision. Int. J. Agric. Biol. Eng..

[24] Chen J., Song J., Guan Z., Lian Y. (2021). Measurement of the distance from grain divider to harvesting boundary based on dynamic regions of interest. Int. J. Agric. Biol. Eng..

[25] Wang Q., Qin W., Liu M., Zhao J., Zhu Q., Yin Y. (2024). Semantic segmentation model-based boundary line recognition method for wheat harvesting. Agriculture.

[26] Zhong W., Yang W., Zhu J., Jia W., Dong X., Ou M. (2024). An Improved UNet-Based Path Recognition Method in Low-Light Environments. Agriculture.

[27] Wang C., Chen X., Jiao Z., Song S., Ma Z. (2025). An improved YOLOP lane-line detection utilizing feature shift aggregation for intelligent agricultural machinery. Agriculture.

[28] Fang H., Hu J., Chen X., Zhang Q., Bai J. (2025). DBSCAN-MFI Based Improved Clustering for Field-Road Classification in Mechanical Residual Film Recovery. Agriculture.

[29] Xu N., Ning X., Li A., Li Z., Song Y., Wu W. (2025). Research on Orchard Navigation Line Recognition Method Based on U-Net. Sensors.

[30] Zhang Q., Xu W., Wu W., Xu L., Zhao Z., Liang S. (2026). Passable area evaluation of tractor road based on improved YOLOv5s and multi-factor fusion. Agriculture.

[31] Zhu L., Deng W., Lai Y., Guo X., Zhang S. (2024). Research on Improved Road Visual Navigation Recognition Method Based on DeepLabV3+ in Pitaya Orchard. Agronomy.

[32] Liu W., Zhou J., Liu Y., Zhang T., Yan M., Chen J., Zhou C., Hu J., Chen X. (2024). An ultrasonic ridge-tracking method based on limiter sliding window filter and fuzzy pure pursuit control for ridge transplanter. Agriculture.

[33] Liu C., Xie S., Sui X., Huang Y., Ma X., Guo N., Yang F. (2023). PRM-D* method for mobile robot path planning. Sensors.

[34] Ma Z., Wang X., Chen X., Hu B., Li J. (2025). Advances in Crop Row Detection for Agricultural Robots: Methods, Performance Indicators, and Scene Adaptability. Agriculture.

[35] Zhou J., Geng S., Qiu Q., Shao Y., Zhang M. (2022). A deep-learning extraction method for orchard visual navigation lines. Agriculture.

[36] Jia W., Zheng Y., Zhao D.A., Yin X., Liu X., Du R. (2018). Preprocessing method of night vision image application in apple harvesting robot. Int. J. Agric. Biol. Eng..

[37] Hu T., Wang W., Gu J., Xia Z., Zhang J., Wang B. (2023). Research on apple object detection and localization method based on improved YOLOX and RGB-D images. Agronomy.

[38] Zhang Z., Lu Y., Zhao Y., Pan Q., Jin K., Xu G., Hu Y. (2023). Ts-yolo: An all-day and lightweight tea canopy shoots detection model. Agronomy.

[39] Radcliffe J., Cox J., Bulanon D.M. (2018). Machine vision for orchard navigation. Comput. Ind..

[40] Blok P.M., van Boheemen K., van Evert F.K., IJsselmuiden J., Kim G.H. (2019). Robot navigation in orchards with localization based on Particle filter and Kalman filter. Comput. Electron. Agric..

[41] Opiyo S., Okinda C., Zhou J., Mwangi E., Makange N.R. (2021). Medial axis-based machine-vision system for orchard robot navigation. Comput. Electron. Agric..

[42] Huang P., Huang P., Wang Z., Wu X., Liu J., Zhu L. (2023). Deep-learning-based trunk perception with depth estimation and DWA for robust navigation of robotics in orchards. Agronomy.

[43] Gu B., Liu Q., Gao Y., Tian G., Zhang B., Wang H., Li H. (2023). Research on the Relative Position Detection Method between Orchard Robots and Fruit Tree Rows. Sensors.

[44] Tang S., Xia Z., Gu J., Wang W., Huang Z., Zhang W. (2024). High-precision apple recognition and localization method based on RGB-D and improved SOLOv2 instance segmentation. Front. Sustain. Food Syst..

[45] Zheng K., Yang S., Wang Z., Fu H., Wang X., Zou W., Zhai C., Chen L. (2026). Real-time detection and validation of a target-oriented model for spindle-shaped tree trunks leveraging deep learning. Agronomy.

[46] Devanna R.P., Milella A., Marani R., Garofalo S.P., Vivaldi G.A., Pascuzzi S., Galati R., Reina G. (2022). In-field automatic identification of pomegranates using a farmer robot. Sensors.

[47] Memon M.S., Chen S., Shen B., Liang R., Tang Z., Wang S., Zhou W., Memon N. (2025). Automatic visual recognition, detection and classification of weeds in cotton fields based on machine vision. Crop Prot..

[48] Syed T.N., Zhou J., Lakhiar I.A., Marinello F., Gemechu T.T., Rottok L.T., Jiang Z. (2025). Enhancing autonomous orchard navigation: A real-time convolutional neural network-based obstacle classification system for distinguishing ‘real’ and ‘fake’ obstacles in agricultural robotics. Agriculture.

[49] Liu Z., Wu J., Cai Y., Wang H., Chen L., Liu Q. (2025). Dual-stage feature specialization network for robust visual object detection in autonomous vehicles. Sci. Rep..

[50] Inoue K., Kaizu Y., Igarashi S., Furuhashi K., Imou K. (2022). Autonomous navigation and obstacle avoidance in an orchard using machine vision techniques for a robotic mower. Eng. Agric. Environ. Food.

[51] Navone A., Martini M., Ambrosio M., Ostuni A., Angarano S., Chiaberge M. (2025). GPS-free autonomous navigation in cluttered tree rows with deep semantic segmentation. Robot. Auton. Syst..

[52] Wang J., Yang L., Cen H., He Y., Liu Y. (2025). Dynamic obstacle avoidance control based on a novel dynamic window approach for agricultural robots. Comput. Ind..

[53] Jiang Q., Shen Y., Liu H., Khan Z., Sun H., Huang Y. (2025). A hybrid path planning algorithm for orchard robots based on an improved D* Lite algorithm. Agriculture.

[54] He Q., Wang Z., Li K., Zhang Y., Li M. (2025). Research on autonomous navigation of mobile robots based on IA-DWA algorithm. Sci. Rep..

[55] Katona K., Neamah H.A., Korondi P. (2024). Obstacle avoidance and path planning methods for autonomous navigation of mobile robot. Sensors.

[56] Niu J., Shen C., Zhang L., Gao G., Zheng J. (2025). Study on Multiobjective Path Optimization of Plant Protection Robots Based on Improved ACO-DWA Algorithm. Eng. Rep..

[57] Wu H., Zhang Y., Huang L., Zhang J., Luan Z., Zhao W., Chen F. (2023). Research on vehicle obstacle avoidance path planning based on APF-PSO. Proc. Inst. Mech. Eng. Part D J. Automob. Eng..

[58] Yang D., Dong L., Dai J.K. (2024). Collision avoidance trajectory planning for a dual-robot system: Using a modified APF method. Robotica.

[59] Zhuang X., Li Y. (2023). Segmentation and angle calculation of rice lodging during harvesting by a combine harvester. Agriculture.

[60] Chatzisavvas A., Dossis M., Dasygenis M. (2024). Optimizing mobile robot navigation based on A-star algorithm for obstacle avoidance in smart agriculture. Electronics.

[61] Ma J., Zhao Y., Fan W., Liu J. (2024). An improved YOLOv8 model for lotus seedpod instance segmentation in the lotus pond environment. Agronomy.

[62] Feng G., Wang C., Wang A., Gao Y., Zhou Y., Huang S., Luo B. (2024). Segmentation of wheat lodging areas from UAV imagery using an ultra-lightweight network. Agriculture.

[63] LaValle S.M., Kuffner J.J., Donald B.R., Lynch K.M., Rus D. (2001). Rapidly-exploring random trees: Progress and prospects. Algorithmic and Computational Robotics: New Directions.

[64] Karaman S., Frazzoli E. (2011). Sampling-based algorithms for optimal motion planning. Int. J. Robot. Res..

[65] Hao K., Zhao J., Yu K., Li C., Wang C. (2020). Path planning of mobile robots based on a multi-population migration genetic algorithm. Sensors.

[66] Chi Z., Yu Z., Wei Q., He Q., Li G., Ding S. (2023). High-efficiency navigation of nonholonomic mobile robots based on improved hybrid A* algorithm. Appl. Sci..

[67] Xu X., Zeng J., Zhao Y., Lü X. (2024). Research on global path planning algorithm for mobile robots based on improved A*. Expert Syst. Appl..

[68] Li X., Wang L., An Y., Huang Q.-L., Cui Y.-H., Hu H.-S. (2024). Dynamic path planning of mobile robots using adaptive dynamic programming. Expert Syst. Appl..

[69] Meng T., Yang T., Huang J., Jin W., Zhang W., Jia Y., Wan K., Xiao G., Yang D., Zhong Z. (2023). Improved hybrid A-star algorithm for path planning in autonomous parking system based on multi-stage dynamic optimization. Int. J. Automot. Technol..

[70] Das M.S., Sanyal S., Mandal S. (2022). Navigation of multiple robots in formative manner in an unknown environment using artificial potential field based path planning algorithm. Ain Shams Eng. J..

[71] Mahmud M.S.A., Abidin M.S.Z., Mohamed Z., Abd Rahman M.K.I., Iida M. (2019). Multi-objective path planner for an agricultural mobile robot in a virtual greenhouse environment. Comput. Electron. Agric..

[72] Wang X., Wei J., Zhou X., Xia Z., Gu X. (2022). AEB-RRT*: An adaptive extension bidirectional RRT* algorithm. Auton. Robots.

[73] Pei H., Sun Y., Huang H., Zhang W., Sheng J., Zhang Z. (2022). Weed detection in maize fields by UAV images based on crop row preprocessing and improved YOLOv4. Agriculture.

[74] Zhang F., Chen Z., Ali S., Yang N., Fu S., Zhang Y. (2023). Multi-class detection of cherry tomatoes using improved YOLOv4-Tiny. Int. J. Agric. Biol. Eng..

[75] Wang J., Gao Z., Zhang Y., Zhou J., Wu J., Li P. (2022). Real-time detection and location of potted flowers based on a ZED camera and a YOLO V4-tiny deep learning algorithm. Horticulturae.

[76] Zhao Y., Zhang X., Sun J., Yu T., Cai Z., Zhang Z., Mao H. (2024). Low-cost lettuce height measurement based on depth vision and lightweight instance segmentation model. Agriculture.

[77] Sun J., He X., Ge X., Wu X., Shen J., Song Y. (2018). Detection of key organs in tomato based on deep migration learning in a complex background. Agriculture.

[78] Liu H., Zeng X., Shen Y., Xu J., Khan Z. (2025). A single-stage navigation path extraction network for agricultural robots in orchards. Comput. Electron. Agric..

[79] Affonso F., Tommaselli F.A.G., Capezzuto G., Gasparino M.V., Chowdhary G., Becker M. (2025). CROW: A self-supervised crop row navigation algorithm for agricultural fields. J. Intell. Robot. Syst..

[80] Xue X., Luo Q., Bu M., Li Z., Lyu S., Song S. (2023). Citrus tree canopy segmentation of orchard spraying robot based on RGB-D image and the improved DeepLabv3+. Agronomy.

[81] Zhang X., Zhou Z., Liu Z. (2026). A Deep Learning-Driven Binocular Vision Path Detection Approach for Orchard Robots. AgriEngineering.

[82] Luo Y., Wei L., Xu L., Zhang Q., Liu J., Cai Q., Zhang W. (2022). Stereo-vision-based multi-crop harvesting edge detection for precise automatic steering of combine harvester. Biosyst. Eng..

[83] Zhu F., Chen J., Guan Z., Zhu Y., Shi H., Cheng K. (2024). Development of a combined harvester navigation control system based on visual simultaneous localization and mapping-inertial guidance fusion. J. Agric. Eng..

[84] Guan X., Ge H., Nie S., Ding Y. (2025). Research on agricultural autonomous positioning and navigation system based on LIO-SAM and Apriltag fusion. Agronomy.

[85] Ma Z., Yang S., Li J., Qi J. (2024). Research on slam localization algorithm for orchard dynamic vision based on YOLOD-SLAM2. Agriculture.

[86] Li Y., Feng Q., Ji C., Sun J., Sun Y. (2024). GNSS and LiDAR Integrated Navigation Method in Orchards with Intermittent GNSS Dropout. Appl. Sci..

[87] Brown J., Paudel A., Biehler D., Thompson A., Karkee M., Grimm C., Davidson J.R. (2024). Tree detection and in-row localization for autonomous precision orchard management. Comput. Electron. Agric..

[88] Liu H., Gu H., Wang Y., Zhong T., Tian T., Geng C. (2026). A GNSS–Vision Integrated Autonomous Navigation System for Trellis Orchard Transportation Robots. AI.

[89] Oh S., Jeon H.-Y., Seo J., Choi K.H., Kim K.-S. (2026). Field-validated navigation framework for orchard weeding robots using multimodal sensor fusion. Int. J. Control Autom. Syst..

[90] Li Z., Liu X., Wang H., Song J., Xie F., Wang K. (2024). Research on robot path planning based on point cloud map in orchard environment. IEEE Access.

[91] Liu Z., Fang J., Zhao Y. (2025). DynaFusion-SLAM: Multi-Sensor Fusion and Dynamic Optimization of Autonomous Navigation Algorithms for Pasture-Pushing Robot. Sensors.

[92] Su Z., Zou W., Zhai C., Tan H., Yang S., Qin X. (2024). Design of an Autonomous Orchard Navigation System Based on Mul-ti-Sensor Fusion. Agronomy.

[93] Chen Z., Dou H., Gao Y., Zhai C., Wang X., Zou W. (2025). Research on an orchard row centreline multipoint autonomous navigation method based on LiDAR. Artif. Intell. Agric..

[94] Hussain I., Han X., Ha J.-W. (2025). Stereo Visual Odometry and Real-Time Appearance-Based SLAM for Mapping and Localization in Indoor and Outdoor Orchard Environments. Agriculture.

[95] Bai Q. (2025). Deep reinforcement learning-driven decision support system design for precision agriculture: Optimizing farming and resource use. Turk. J. Agric. For..

[96] Chen W.H. (2022). Perspective view of autonomous control in unknown environment: Dual control for exploitation and exploration vs reinforcement learning. Neurocomputing.

[97] Chen Y., Fu Y., Zhang B., Fu W., Shen C. (2022). Path planning of the fruit tree pruning manipulator based on improved RRT-Connect algorithm. Int. J. Agric. Biol. Eng..

[98] Li Y., Li J., Zhou W., Yao Q., Nie J., Qi X. (2022). Robot path planning navigation for dense planting red jujube orchards based on the joint improved A* and DWA algorithms under laser SLAM. Agriculture.

[99] Xu Y., Xue X., Sun Z., Gu W., Cui L., Jin Y., Lan Y. (2025). Global path planning for navigating orchard vehicle based on fruit tree positioning and planting rows detection from UAV imagery. Comput. Electron. Agric..

[100] Hizatate T., Noguchi N. (2023). Work schedule optimization for electric agricultural robots in orchards. Comput. Electron. Agric..

[101] Xie J., Liu S., Wang X., Liu L., Wang X., Li J., Yang X. (2025). Collaborative path planning and task allocation for multiple mowing robots in the standard orchards. Int. J. Agric. Biol. Eng..

[102] Husain Z., Al Zaabi A., Hildmann H., Saffre F., Ruta D., Isakovic A. (2022). Search and rescue in a maze-like environment with ant and Dijkstra algorithms. Drones.

[103] Jin P., Li T., Pan Y., Hu K., Xu N., Ying W., Jin Y., Kang H. (2024). A Context-Aware Navigation Framework for Ground Robots in Horticultural Environments. Sensors.

[104] Li T., Guo Y., Liu H., Shen Y., Shen Y. (2025). Real-time local trajectory optimization of an orchard robot using improved ALTRO. Trans. Chin. Soc. Agric. Eng..

[105] Li Z., Chen W.H., Yang J., Liu C. (2024). Cooperative active learning-based dual control for exploration and exploitation in autonomous search. IEEE Trans. Neural Netw. Learn. Syst..

[106] Li Z., Chen W.H., Yang J., Yan Y. (2024). Dual control of exploration and exploitation for auto-optimization control with active learning. IEEE Trans. Autom. Sci. Eng..

[107] Liu L., Wang X., Xie J., Wang X., Liu H., Li J., Wang P., Yang X. (2025). Path planning and tracking control of orchard wheel mower based on BL-ACO and GO-SMC. Comput. Electron. Agric..

[108] Lu E., Ma Z., Li Y., Xu L., Tang Z. (2020). Adaptive backstepping control of tracked robot running trajectory based on real-time slip parameter estimation. Int. J. Agric. Biol. Eng..

[109] Yu Y., Liu Y., Wang J., Noguchi N., He Y. (2023). Obstacle avoidance method based on double DQN for agricultural robots. Comput. Electron. Agric..

[110] Hu M., Huang Q., Cai J., Chen Y., Li J., Shi L. (2025). HPS-RRT*: An improved path planning algorithm for a nonholonomic orchard robot in unstructured environments. Agronomy.

[111] Shih C.H., Lin C.J., Jhang J.Y. (2023). Ackerman unmanned mobile vehicle based on heterogeneous sensor in navigation control application. Sensors.

[112] Vashisth A., Rückin J., Magistri F., Stachniss C., Popović M. (2024). Deep reinforcement learning with dynamic graphs for adaptive informative path planning. IEEE Robot. Autom. Lett..

[113] Zhu X., Zhao X., Liu J., Feng W., Fan X. (2025). Autonomous navigation and obstacle avoidance for orchard spraying robots: A sensor-fusion approach with ArduPilot, ROS, and EKF. Agronomy.

[114] Zhang W., Zeng Y., Wang S., Wang T., Li H., Fei K., Qiu X., Jiang R., Li J. (2023). Research on the local path planning of an orchard mowing robot based on an elliptic repulsion scope boundary constraint potential field method. Front. Plant Sci..

[115] Wang Y., Fu C., Huang R., Tong K., He Y., Xu L. (2024). Path planning for mobile robots in greenhouse orchards based on improved A* and fuzzy DWA algorithms. Comput. Electron. Agric..

[116] Cui L., Le F., Xue X., Sun T., Jiao Y. (2024). Design and experiment of an agricultural field management robot and its navigation control system. Agronomy.

[117] Xu L., You J., Yuan H. (2023). Real-time parametric path planning algorithm for agricultural machinery kinematics model based on particle swarm optimization. Agriculture.

[118] Wang M., Xu J., Zhang J., Cui Y. (2024). An autonomous navigation method for orchard rows based on a combination of an improved A-star algorithm and SVR. Precis. Agric..

[119] Ye L., Wu F., Zou X., Li J. (2023). Path planning for mobile robots in unstructured orchard environments: An improved kinematically constrained bi-directional RRT approach. Comput. Electron. Agric..

[120] Zhang M., Li X., Wang L., Jin L., Wang S. (2024). A path planning system for orchard mower based on improved A* algorithm. Agronomy.

[121] Ye L., Li J., Li P. (2024). Improving path planning for mobile robots in complex orchard environments: The continuous bidirectional Quick-RRT* algorithm. Front. Plant Sci..

[122] Zheng S., Xu S., Rai R. (2025). Trailer-referenced autonomous navigation of agricultural tractor-trailer systems. Comput. Electron. Agric..

[123] Raikwar S., Fehrmann J., Herlitzius T. (2022). Navigation and control development for a four-wheel-steered mobile orchard robot using model-based design. Comput. Electron. Agric..

[124] Shen Y., Shen Y., Zhang Y., Huo C., Shen Z., Su W., Liu H. (2025). Research progress on path planning and tracking control methods for orchard mobile robots in complex scenarios. Agriculture.

[125] Al-Jumaili M.H., Özok Y.E. (2024). New control model for autonomous vehicles using integration of Model Predictive and Stanley based controllers. Sci. Rep..

[126] Ding C., Ding S., Wei X., Mei K. (2022). Output feedback sliding mode control for path-tracking of autonomous agricultural vehicles. Nonlinear Dyn..

[127] Farkh R., Aljaloud K. (2023). Vision navigation based PID control for line tracking robot. Intell. Autom. Soft Comput..

[128] Fu W., Liu Y., Zhang X. (2023). Research on accurate motion trajectory control method of four-wheel steering AGV based on Stanley-PID control. Sensors.

[129] Li J., Wang S., Zhang W., Li H., Zeng Y., Wang T., Fei K., Qiu X., Jiang R., Mai C. (2023). Research on Path Tracking for an Orchard Mowing Robot Based on Cascaded Model Predictive Control and Anti-Slip Drive Control. Agronomy.

[130] Cui B., Cui X., Wei X., Zhu Y., Ma Z., Zhao Y., Liu Y. (2024). Design and testing of a tractor automatic navigation system based on dynamic path search and a fuzzy stanley model. Agriculture.

[131] Sun J., Wang Z., Ding S., Xia J., Xing G. (2024). Adaptive disturbance observer-based fixed time nonsingular terminal sliding mode control for path-tracking of unmanned agricultural tractors. Biosyst. Eng..

[132] Wang X., Zhang B., Du X., Chen H., Zhu T., Wu C. (2025). Self-adjusting look-ahead distance of precision path tracking for high-clearance sprayers in field navigation. Agronomy.

[133] Hu C., Ru Y., Li X., Fang S., Zhou H., Yan X., Liu M., Xie R. (2024). Path tracking control for brake-steering tracked vehicles based on an improved pure pursuit algorithm. Biosyst. Eng..

[134] Hu K., Cheng K. (2023). Trajectory planning for an articulated tracked vehicle and tracking the trajectory via an adaptive model predictive control. Electronics.

[135] Li B., Wang Y., Ma S., Bian X., Li H., Zhang T., Li X., Zhang Y. (2023). Adaptive pure pursuit: A real-time path planner using tracking controllers to plan safe and kinematically feasible paths. IEEE Trans. Intell. Veh..

[136] Li X., Liu X., Wang G., Gu K., Che H. (2022). Discrete open-closed-loop PID-type iterative learning control for trajectory tracking of tracked mobile robots. Int. J. Adv. Robot. Syst..

[137] Liu G., Li C., Shen Y. (2021). Trajectory tracking and fuzzy adaptive model predictive control of high clearance synchronous-steering sprayer. Trans. Chin. Soc. Agric. Mach..

[138] Liu H., Yan S., Shen Y., Li C., Zhang Y., Hussain F. (2021). Model predictive control system based on direct yaw moment control for 4WID self-steering agriculture vehicle. Int. J. Agric. Biol. Eng..

[139] Xu J., Lai J., Guo R., Lu X., Xu L. (2022). Efficiency-oriented MPC algorithm for path tracking in autonomous agricultural machinery. Agronomy.

[140] Ma B., Pei W., Zhang Q. (2023). Trajectory tracking control of autonomous vehicles based on an improved sliding mode control scheme. Electronics.

[141] Ma J., Xie H., Song K., Liu H. (2022). Self-Optimizing Path Tracking Controller for Intelligent Vehicles Based on Reinforcement Learning. Symmetry.

[142] Peicheng S., Li L., Ni X., Yang A. (2022). Intelligent vehicle path tracking control based on improved MPC and hybrid PID. IEEE Access.

[143] Sekban H.T., Basci A. (2023). Designing new model-based adaptive sliding mode controllers for trajectory tracking control of an unmanned ground vehicle. IEEE Access.

[144] Song Y., Xue J., Zhang T., Sun X., Sun H., Gao W., Chen Q. (2025). Path tracking control of crawler tractor based on adaptive adjustment of lookahead distance using sparrow search algorithm. Comput. Electron. Agric..

[145] Comelli R., Olaru S., Seron M.M., Kofman E. (2024). Application of a stabilizing model predictive controller to path following for a car-like agricultural robot. Optim. Control Appl. Methods.

[146] Sun Y., Cui B., Ji F., Wei X., Zhu Y. (2022). The full-field path tracking of agricultural machinery based on PSO-enhanced fuzzy Stanley model. Appl. Sci..

[147] Zhu C.W., Hill E., Biglarbegian M., Gadsden S.A., Cline J.A. (2023). Smart agriculture: Development of a skid-steer autonomous robot with advanced model predictive controllers. Robot. Auton. Syst..

[148] Wang L., Zhai Z., Zhu Z., Mao E. (2022). Path tracking control of an autonomous tractor using improved Stanley controller optimized with multiple-population genetic algorithm. Actuators.

[149] Wang P., Gao S., Li L., Cheng S., Zhao L. (2019). Automatic steering control strategy for unmanned vehicles based on robust backstepping sliding mode control theory. IEEE Access.

[150] Wang S., Guo J., Mao Y., Wang H., Fan J. (2023). Research on the model predictive trajectory tracking control of unmanned ground tracked vehicles. Drones.

[151] Lu E., Xue J., Chen T., Jiang S. (2023). Robust trajectory tracking control of an autonomous tractor-trailer considering model parameter uncertainties and disturbances. Agriculture.

[152] Wen J., Yao L., Zhou J., Yang Z., Xu L., Yao L. (2025). Path Tracking Control of Agricultural Automatic Navigation Vehicles Based on an Improved Sparrow Search-Pure Pursuit Algorithm. Agriculture.

[153] Zhou B., Su X., Yu H., Guo W., Zhang Q. (2023). Research on path tracking of articulated steering tractor based on modified model predictive control. Agriculture.

[154] Yan X., Wang S., He Y., Ma A., Zhao S. (2025). Autonomous Tracked Vehicle Trajectory Tracking Control Based on Disturbance Observation and Sliding Mode Control. Actuators.

[155] Yang Y., Li Y., Wen X., Zhang G., Ma Q., Cheng S., Qi J., Xu L., Chen L. (2022). An optimal goal point determination algorithm for automatic navigation of agricultural machinery: Improving the tracking accuracy of the Pure Pursuit algorithm. Comput. Electron. Agric..

[156] Wang X., Zhang B., Du X., Hu X., Wu C., Cai J. (2025). An Adaptive Path Tracking Controller with Dynamic Look-Ahead Distance Optimization for Crawler Orchard Sprayers. Actuators.

[157] Zhang S., Wei X., Liu C., Ge J., Cui X., Wang F., Wang A., Chen W. (2025). Adaptive path tracking and control system for unmanned crawler harvesters in paddy fields. Comput. Electron. Agric..

[158] Zhang L., Zhang R., Zhang D., Yi T., Ding C., Chen L. (2025). Path curvature incorporated reinforcement learning method for accurate path tracking of agricultural vehicles. Comput. Electron. Agric..

[159] Zhou J., Wen J., Yao L., Yang Z., Xu L., Yao L. (2025). Agricultural machinery path tracking with varying curvatures based on an improved pure-pursuit method. Agriculture.

[160] Zou T., Angeles J., Hassani F. (2018). Dynamic modeling and trajectory tracking control of unmanned tracked vehicles. Robot. Auton. Syst..

[161] Zheng K., Zhao X., Han C., He Y., Zhai C., Zhao C. (2023). Design and experiment of an automatic row-oriented spraying system based on machine vision for early-stage maize corps. Agriculture.

[162] Huang S., Pan K., Wang S., Zhu Y., Zhang Q., Su X., Yu H. (2023). Design and test of an automatic navigation fruit-picking platform. Agriculture.

[163] Jiang A., Ahamed T. (2023). Navigation of an autonomous spraying robot for orchard operations using LiDAR for tree trunk detection. Sensors.

[164] Liu H., Du Z., Shen Y., Du W., Zhang X. (2024). Development and evaluation of an intelligent multivariable spraying robot for orchards and nurseries. Comput. Electron. Agric..

[165] Wang Y., Liu D., Zhao H., Li Y., Song W., Liu M., Tian L., Yan X. (2022). Rapid citrus harvesting motion planning with pre-harvesting point and quad-tree. Comput. Electron. Agric..

[166] Chen K., Li T., Yan T., Xie F., Feng Q., Zhu Q., Zhao C. (2022). A soft gripper design for apple harvesting with force feedback and fruit slip detection. Agriculture.

[167] Gao R., Zhou Q., Cao S., Jiang Q. (2023). Apple-picking robot picking path planning algorithm based on improved PSO. Electronics.

[168] Guo Z., Fu H., Wu J., Han W., Huang W., Zheng W., Li T. (2025). Dynamic Task Planning for Multi-Arm Apple-Harvesting Robots Using LSTM-PPO Reinforcement Learning Algorithm. Agriculture.

[169] Tang Z., Xu L., Wang Y., Kang Z., Xie H. (2021). Collision-free motion planning of a six-link manipulator used in a citrus picking robot. Appl. Sci..

[170] Zhang B., Yin C., Fu Y., Xia Y., Fu W. (2024). Harvest motion planning for mango picking robot based on improved RRT-Connect. Biosyst. Eng..

[171] Dong X., Kim W.Y., Yu Z., Oh J.Y., Ehsani R., Lee K.H. (2024). Improved voxel-based volume estimation and pruning severity mapping of apple trees during the pruning period. Comput. Electron. Agric..

[172] Kong F., Liu B., Han X., Yi L., Sun H., Liu J., Liu L., Lan Y. (2024). Path Planning Algorithm of Orchard Fertilization Robot Based on Multi-Constrained Bessel Curve. Agriculture.

[173] Li Y., Ma S. (2023). Navigation of apple tree pruning robot based on improved RRT-connect algorithm. Agriculture.

[174] Shen Y., Liu Z., Liu H., Du W. (2023). Orchard spray robot planning algorithm based on multiple constraints. Trans. Chin. Soc. Agric. Mach..

[175] Tian H., Mo Z., Ma C., Xiao J., Jia R., Lan Y., Zhang Y. (2023). Design and validation of a multi-objective waypoint planning algorithm for UAV spraying in orchards based on improved ant colony algorithm. Front. Plant Sci..

[176] Yang T., Du X., Zhang B., Wang X., Zhang Z., Wu C. (2025). Coverage path planning based on region segmentation and path orientation optimization. Agriculture.

[177] Li S., Zhang M., Wang N., Cao R., Zhang Z., Ji Y., Li H., Wang H. (2023). Intelligent scheduling method for multi-machine cooperative operation based on NSGA-III and improved ant colony algorithm. Comput. Electron. Agric..

[178] Wang Y., He Z., Cao D., Ma L., Li K., Jia L., Cui Y. (2023). Coverage path planning for kiwifruit picking robots based on deep reinforcement learning. Comput. Electron. Agric..

[179] Xu Y., Xue X., Sun Z., Cui L., Cai C., Zhou Q., Chen C., Gu W. (2025). Path planning and scheduling of multiple unmanned ground vehicles for orchard plant protection operations. Comput. Electron. Agric..

[180] Zhang M., Liu P., Li J., Liu L., Zhang H., Yang L. (2024). Local path planning method based on smooth time elastic band algorithm for orchard robotic lawn mower. INMATEH-Agric. Eng..

[181] Chen J., Lian Y., Zou R., Zhang S., Ning X., Han M. (2020). Real-time grain breakage sensing for rice combine harvesters using machine vision technology. Int. J. Agric. Biol. Eng..

